# Functional TFEB activation characterizes multiple models of renal cystic disease and loss of polycystin-1

**DOI:** 10.1152/ajprenal.00237.2022

**Published:** 2023-02-16

**Authors:** Jonathan M. Shillingford, James A. Shayman

**Affiliations:** Department of Internal Medicine, University of Michigan Medical School, University of Michigan, Ann Arbor, Michigan, United States

**Keywords:** autophagic flux, glycoprotein nonmetastatic melanoma protein B, lysosome, polycystic kidney, transcription factor EB

## Abstract

Polycystic kidney disease is a disorder of renal epithelial growth and differentiation. Transcription factor EB (TFEB), a master regulator of lysosome biogenesis and function, was studied for a potential role in this disorder. Nuclear translocation and functional responses to TFEB activation were studied in three murine models of renal cystic disease, including knockouts of *folliculin*, *folliculin interacting proteins 1* and *2*, and *polycystin-1* (*Pkd1*) as well as in mouse embryonic fibroblasts lacking *Pkd1* and three-dimensional cultures of Madin-Darby canine kidney cells. Nuclear translocation of Tfeb characterized cystic but not noncystic renal tubular epithelia in all three murine models as both an early and sustained response to cyst formation. Epithelia expressed elevated levels of Tfeb-dependent gene products, including cathepsin B and glycoprotein nonmetastatic melanoma protein B. Nuclear Tfeb translocation was observed in mouse embryonic fibroblasts lacking *Pkd1* but not wild-type fibroblasts. *Pkd1* knockout fibroblasts were characterized by increased Tfeb-dependent transcripts, lysosomal biogenesis and repositioning, and increased autophagy. The growth of Madin-Darby canine kidney cell cysts was markedly increased following exposure to the TFEB agonist compound C1, and nuclear Tfeb translocation was observed in response to both forskolin and compound C1 treatment. Nuclear TFEB also characterized cystic epithelia but not noncystic tubular epithelia in human patients with autosomal dominant polycystic kidney disease. Noncanonical activation of TFEB is characteristic of cystic epithelia in multiple models of renal cystic disease including those associated with loss of *Pkd1*. Nuclear TFEB translocation is functionally active in these models and may be a component of a general pathway contributing to cystogenesis and growth.

**NEW & NOTEWORTHY** Changes in epithelial cell metabolism are important in renal cyst development. The role of TFEB, a transcriptional regulator of lysosomal function, was explored in several models of renal cystic disease and human ADPKD tissue sections. Nuclear TFEB translocation was uniformly observed in cystic epithelia in each model of renal cystic disease examined. TFEB translocation was functionally active and associated with lysosomal biogenesis and perinuclear repositioning, increased TFEB-associated protein expression, and activation of autophagic flux. Compound C1, a TFEB agonist, promoted cyst growth in 3-D cultures of MDCK cells. Nuclear TFEB translocation is an underappreciated signaling pathway for cystogenesis that may represent a new paradigm for cystic kidney disease.

## Graphical abstract

**Figure:**
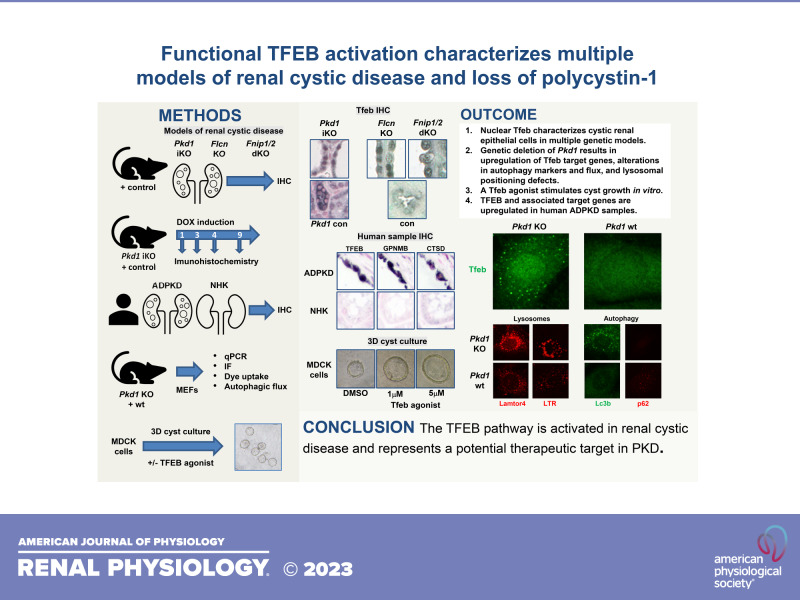

## INTRODUCTION

Autosomal dominant polycystic kidney disease (ADPKD) is among the most common causes of end-stage renal disease in adults. The ADPKD phenotype is characterized by disruption of the tubular diameter of the nephron, excessive tubular epithelial cell growth, fluid secretion, epithelial cell matrix interactions, and macrophage activation (1). A significant body of work has identified several cellular signaling pathways that are either upregulated or downregulated in PKD, pathways largely associated with cAMP and Ca^2+^ signaling, cell proliferation, apoptosis, and fluid secretion (2). Several therapeutic targets have been identified in association with these perturbations, many supported by proof-of-principle studies in animal models of PKD. Presently, only the vasopressin V2 receptor antagonist tolvaptan is approved for clinical use as a specific therapy in humans with ADPKD (3).

Recently, the role of aberrant metabolic regulation as a driver for cystogenesis and growth has been a major focus of investigation. Although oxidative phosphorylation provides much of the energy requirements for differentiated renal tubular epithelial cells, cystic epithelia are characterized by glycolytic flux despite the presence of oxygen: the Warburg effect (4).

The lysosome is a major site for nutrient sensing within cells. In a canonical model of nutrient sensing, mammalian target of rapamycin (mTOR) complex 1 (mTORC1) localizes to the surface of the lysosome and sequesters transcription factor EB (TFEB) within the cell cytosol through phosphorylation. Following nutrient deprivation, TFEB is dephosphorylated and translocated to the nucleus, acting as a master regulator of several compensatory processes including lysosomal biogenesis, lysosomal repositioning and exocytosis, and autophagy. Following nutrient repletion, TFEB egress proceeds and cytosolic sequestration is restored (5).

The plausibility of a role for this transcription factor in ADPKD is supported by the renal cystic phenotypes observed in transgenic mice that overexpress TFEB (6) and in patients with Birt-Hogg-Dubé (BHD) syndrome [for a review, see Daccord et al. (7)], which arises due to mutations in the folliculin (*FLCN*) gene, is associated with Tfeb pathway activation, and is recapitulated in both *Flcn* knockout (KO) (8, 9) and double genetic KO of its interacting proteins, folliculin-interacting proteins 1 and 2 (Fnip1 and Fnip2, respectively) (10), in animal models. These reports beg the question as to whether the cystic phenotype may be a state of nutrient deprivation and, as such, be associated with nuclear TFEB localization.

Using several models of renal cystic disease, including those associated with loss of folliculin and polycystin-1 (PKD1) activities, it was observed that nuclear TFEB translocation and functional responses associated with TFEB activation characterize cyst-lining epithelia. These results provide experimental evidence that the Tfeb pathway is active in cystic epithelia and may be a potential therapeutic target in ADPKD.

## MATERIALS AND METHODS

### Mouse Models

Kidney samples derived from *Flcn^fl/fl^:Ksp-Cre* KO (8) and *Fnip1/2^fl/fl^:Ksp-Cre* double KO (10) mice have been previously described and, along with their respective controls, were kindly provided by Dr. Laura Schmidt (Center for Cancer Research, National Cancer Institute) (10). All renal samples from the *Pkd1^fl/fl^:Pax8-rTTA:TetO-Cre* model were generated by and obtained from the Mouse Models and Biobank Core of the Polycystic Kidney Disease Research Resource Consortium. In brief, this model was generated by crossing B6.129S4-Pkd1^tm2Ggg^/J (RRID: IMSR_JAX:010671) with B6.Cg-Tg(Pax8-rtTA2S*M2)1Koes/J mice (RRID: IMSR_JAX:007176), and renal tissue from these animals was obtained from the Mouse Models and Biobank Core of the Baltimore PKD Research and Clinical Core Center. To study early-onset renal cystogenesis in the *Pkd1^fl/fl^:Pax8-rTTA:TetO-Cre* model, Cre recombinase activity was induced via intraperitoneal administration of 50 µg/kg doxycycline (DOX) hyclate (Cat. No. D9891, Sigma-Aldrich) at *postnatal days 10* and *11* followed by harvesting of kidneys at *postnatal days 12*, *14*, *15*, *20*, and *25*. Control kidneys were obtained from DOX-induced *Pkd1^fl/+^:TetO-Cre* mice lacking *Pax8-rTTA*. To generate a later-onset renal cystic model, DOX was administered at *postnatal days 27–29* and kidneys were harvested at *postnatal day 150*. Subsequently, mice were euthanized with an isoflurane overdose and kidneys were harvested and snap-frozen in liquid nitrogen or processed for histology by immersion in 4% paraformaldehyde (Cat. No. 28908, Invitrogen) overnight at 4°C before being washed three times in ice-cold PBS for at least 30 min each wash before paraffin embedding and sectioning [Rogel Cancer Center Tissue and Molecular Pathology Shared Resource [funding support: National Institutes of Health (NIH) Grant P30CA04659229]. Mice of both sexes were used in this study and are described in Table 1.

**Table 1.** Details of the mouse models and samples used in this study

Model and Genotype	Renal Phenotype	Sex
*Pkd1 iKO*		
P12 (DPD 1), *Pkd1^fl/fl^*:Pax8-rTTa:TetO-Cre	Cystic	One female, two males
P12 (DPD 1), *Pkd1^fl/fl^*:TetO-Cre	Noncystic	One female, two males
P14 (DPD 3), *Pkd1^fl/fl^*:Pax8-rTTa:TetO-Cre	Cystic	One female, two males
P14 (DPD 3), *Pkd1^fl/fl^*:TetO-Cre	Noncystic	Two females, one male
P15 (DPD 4), *Pkd1^fl/fl^*:Pax8-rTTa:TetO-Cre	Cystic	Two females, one male
P15 (DPD 4), *Pkd1^fl/fl^*:TetO-Cre - cystic	Noncystic	Two females, one male
P20 (DPD 9), *Pkd1^fl/fl^*:Pax8-rTTa:TetO-Cre	Cystic	Two females, one male
P20 (DPD 9), *Pkd1^fl/fl^*:TetO-Cre	Noncystic	One female, two males
P25, *Pkd1^fl/fl^*:Pax8-rTTa:TetO-Cre	Cystic	Three males
P25, *Pkd1^fl/fl^*:TetO-Cre	Noncystic	Two females, one male
P150, *Pkd1^fl/fl^*:Pax8-rTTa:TetO-Cre	Cystic	Three males
P150, *Pkd1^fl/fl^*:TetO-Cre	Noncystic	Three males
*Flcn* KO		
P7, *Flcn^fl/fl^*:Ksp-Cre	Cystic	Two females, one male
P7, *Flcn^fl/+^*^:^Ksp-Cre	Noncystic	One female, two males
P14, *Flcn^fl/fl^*:Ksp-Cre	Cystic	One female, two males
P14, *Flcn^fl/+^*^:^Ksp-Cre	Noncystic	Three females
P18, *Flcn^fl/fl^*:Ksp-Cre	Cystic	Two females, one male
P18, *Flcn^fl/+^*^:^Ksp-Cre	Noncystic	Three males
*Fnip1/2* dKO		
P18, *Fnip1^fl/fl^:Fnip2^fl/fl^*:Ksp-Cre	Cystic	Three males
P18, *Fnip1^fl/+^:Fnip2^fl/+^*:Ksp-Cre	Noncystic	Two females, one male

dKO, double knockout; DOX, doxycycline; DPD, days post-DOX; KO, knockout P, postnatal day.

### Human ADPKD Samples

Formalin-fixed paraffin-embedded (FFPE) blocks of normal and ADPKD tissue were obtained from the PKD Biomarkers Core of the Kansas PKD Research and Translation Core Center, an NIH P30-sponsored core, and multiple 5-µm samples were sectioned and placed onto positively charged glass slides [Rogel Cancer Center Tissue and Molecular Pathology Shared Resource (funding support: NIH Grant P30CA04659229)].

### Antibodies

For immunohistochemistry experiments, antibodies against Tfeb/TFEB (Cat. No. A303-673A, Bethyl Laboratories), cathepsin B (CTSB; Cat. No. 31718, Cell Signaling Technologies), cathepsin D (CTSD; Cat. No. 2284, Cell Signaling Technologies), transcription factor E3 (TFE3; Cat. No. PA5-54909, Invitrogen), phospho-rpS6:Ser^235/236^ (Cat. No. 4858, Cell Signaling Technologies), and glycoprotein nonmetastatic melanoma protein B (GPNMB; Cat. No. PA5-42585, Thermo Fisher Scientific) were used. For immunocytochemistry experiments, TFEB (Cat. No. PA1-31552, Invitrogen), late endosomal/lysosomal adaptor and MAPK and mTOR activator 4 (LAMTOR4; Cat. No. 13140, Cell Signaling Technologies), light chain 3B (LC3B; Cat. No. ab192890, Abcam), and sequestosome 1 (SQSTM1)/p62 (Cat. No. 23214, Cell Signaling Technologies) were used. For three-dimensional (3-D) cyst culture experiments, TFEB (Cat. No. A303-673A, Bethyl Laboratories) was used.

Fluorescent-conjugated secondary antibodies were purchased from Abcam (Gt α rabbit DyLight 488, Cat. No. ab96899; Gt α mouse DyLight 488, Cat. No. ab96879; Gt α mouse DyLight 550, Cat. No. ab96872; and Gt α rabbit DyLight 550, Cat. No. ab96884).

### Cell Culture

Madin-Darby canine kidney (MDCK) cells were routinely cultured in MEM (Cat. No. 11090081, Gibco) supplemented with 5% FBS (Cat. No. 16140071, Gibco) and 1× penicillin, streptomycin, and glutamine (Cat. No. 10378016, Gibco). Primary *Pkd1* wild-type and *Pkd1* KO mouse embryonic fibroblasts (MEFs) isolated from *embryonic day* 12.5 *Pkd1* KO and wild-type embryos according to standard protocols were obtained from the Mouse Models and Biobank Core of the Polycystic Kidney Disease Research Resource Consortium. For growth in vitro, MEFs were routinely cultured in DMEM (Cat. No. 11960044, Gibco) supplemented with 10% heat-inactivated FBS and 1× penicillin, streptomycin, and glutamine. Cells were routinely passaged using TrypLE Express (Cat. No. 12605010, Gibco), and the experiments described were performed up to *passage 4*.

### Cyst Culture Growth

For the generation of 3-D cysts, cells between 60% and 80% confluency were detached from a T75 flask and subsequently counted (Corning Cell Counter, CytoSmart). Cells (40,000) and media were mixed in a volume of 200 µL to which 300-µL ice-cold PureCol EZ gel (Cat. No. 5074, Sigma, 3 mg/mL collagen final) and 25 µL/well (2,000 cells) plated in prewarmed angiogenesis slides (Cat. No. 81506, ibidi). The hydrogel matrix was allowed to firm for 2 h at 37°C before the addition of 35-µL media on top of the gel, which was changed every other day. Five days postplating, cyst development was followed under an inverted microscope equipped with phase contrast and images were acquired. Representative images of cysts that had a single, clearly identifiable lumen were acquired (5 images per well, 15 wells) to calculate the average cyst diameter before drug treatment. On *day 5*, cyst cultures were subjected to drug treatment with DMSO, 1 or 5 µM compound C1 (Cat. No. S6769, Selleck Chemicals), or 10 µM forskolin (Cat. No. F3917, Millipore Sigma) in triplicate every day for 4 days. On *day 9*, phase-contrast images (5 cysts per well in triplicate wells for each treatment condition) were acquired before processing for immunocytochemistry.

### Cyst Culture Immunocytochemistry

Cysts were washed three times with PBS (pH 7.4) supplemented with 0.9 mM calcium and 0.5 mM magnesium (PBS+). To improve the permeability of the antibody through the collagen matrix, cyst cultures were incubated with 100 units collagenase (1,000-unit PBS+ stock diluted 1:10 in PBS+) for 10 min at 37°C followed by being washed three times with PBS+. Cysts were fixed with 4% paraformaldehyde in PBS while being shaken on a platform shaker for 30 min at room temperature. Fixed cysts were subsequently washed three times with PBS+ followed by three washes in PBS+ for 10 min. Blocking and permeabilization buffer (BPB) was added to the cyst cultures and incubated for 30 min at room temperature with gentle shaking. Primary antibodies were diluted in BPB and added to the cysts, which were incubated at 4°C overnight in a humidified chamber. The following day, cysts were washed three times with PBS+ followed by three washes in PBS+ for 10 min. For detection, secondary antibodies were diluted in BPB supplemented with NucBlue Live and incubated for 3 h at room temperature with shaking. Subsequently, cysts were washed three times with PBS+ followed by three washes in PBS+ for 15 min. One drop of ProLong Diamond antifade mounting media (Cat. No. P36970, Invitrogen) was added and allowed to solidify overnight before imaging and analysis.

### Autophagic Flux Reporter Assays

*Pkd1* KO and wild-type MEF cells (∼70% confluent) plated in eight-well chamber slides were incubated with 0.3 mL media containing 10 µL/well Premo Autophagy Tandem Sensor red fluorescent protein (RFP)-green fluorescent protein (GFP)-LC3B (Cat. No. P36239, Invitrogen) for 24 h at 37°C in a cell culture incubator. To increase transduction efficiency, the sensor was removed and replaced with fresh media supplemented with 1:1,000 BacMam enhancer reagent (Cat. No. B10107, Invitrogen) and incubated for 2 h at 37°C in a cell culture incubator before replacement with fresh media and overnight incubation at 37°C. The following day, media were replaced with fresh media containing either 0.1% DMSO (control) or 200 nM bafilomycin A1 (Cat. No. HY-100558, MedChemExpress) for 4 h, and cells were subsequently washed with FluoroBrite DMEM. The media were replaced with FluoroBrite DMEM + 10% FBS, and cells were subjected to live cell imaging analysis on a Leica SP8 confocal microscope using the appropriate wavelength settings. To determine colocalization, separated red and green channels were subjected to identical background subtraction (rolling ball radius of 10 pixels, sliding paraboloid) in ImageJ, and colocalized pixels were determined using the Colocalization Threshold plugin.

### Lysosome Dye Uptake Assays

Following cell plating and establishment of adherence in eight-well chamber slides, 50 nM LysoTracker red (Cat. No. L7528, Invitrogen) or 1:1,000 dilution of acridine orange (Cat. No. 6130, Immunochemistry Technologies) and one drop of NucBlue Live (Cat. No. R37605, Invitrogen) were added to the cells, which were subsequently incubated for 30 min at 37°C in a cell culture incubator to permit cellular uptake. Following incubation, cells were washed in FluroBrite DMEM (Cat. No. A1896702, Gibco) and imaged live without fixation in FluroBrite DMEM supplemented with 10% FBS.

### Immunostaining

For cellular immunocytochemistry experiments, cells were grown in eight-well culture slides to the desired confluency. The cell culture media were removed from each well, and cells were washed twice with 0.5 mL of 1× D-PBS. Cells were fixed with 4% paraformaldehyde [16% diluted in 3 volumes of 1× PBS (pH 7.4), Cat. No. 43368, Alfa Aesar] for 15 min at room temperature, washed twice with 0.5 mL of 1× PBS (pH 7.4), and incubated for 30 min at 37°C with 0.5 mL BPB (3% normal goat or donkey serum, 1% BSA, 0.1% Triton X-100, and 0.05% Tween 20 in 1× PBS, pH 7.4). Cells were washed twice with 1× PBS (pH 7.4) and incubated with various primary antibodies (see Table 2) diluted in primary antibody dilution buffer (2% BSA and 0.5% Triton X-100 in 1× PBS, pH 7.4) overnight at 4°C in a humidified chamber. The following day, cells were washed twice with 0.5 mL of 1× PBS (pH 7.4) and incubated with 150 µL/well of fluorescent-conjugated secondary antibodies (see Table 2) diluted in secondary antibody dilution buffer (0.05% Tween 20 in 1× PBS, pH 7.4) for 1 h at 37°C in a humidified chamber. Cells were washed twice with 0.5 mL of 1× PBS (pH 7.4) and postfixed for 10 min with 0.5 mL of 4% paraformaldehyde solution. Cells were washed twice with 0.5 mL of 1× PBS (pH 7.4), and one drop of NucBlue Live (Cat. No. R37605, Invitrogen) was applied to each well and incubated for 20 min at room temperature in the dark. Cells were washed once with 0.5 mL of 1× PBS (pH 7.4), and slides were mounted with ProLong Diamond anti-fade mounting reagent (Cat. No. P36970, Invitrogen).

**Table 2.** Details of antibodies and reagents used in this study

Reagent	Supplier	Cat. No.	Dilution	Assay
Primary antibody				
phospho-rpS6 (Ser235/6)	CST	4858	1:200	IHC
CTSB	CST	31718	1:200	IHC
TFEB	Bethyl Laboratories	A303-673A	1:1,000	IHC, 3-D cyst
TFEB	Bethyl Laboratories	A303-673A	1:200	IF (FFPE)
TFE3	Invitrogen	PA5-54909	1:200	IHC
GPNMB	Invitrogen	PA5-42585	1:100	IHC
CTSD	CST	2284	1:200	IHC
TFEB	Invitrogen	PA1-31552	1:200	IF (MDCK)
LAMTOR4	CST	13140	1:200	IF
LC3B	Abcam	ab192890	1:200	IF
SQSTM1/p62	CST	23214	1:200	IF
Secondary antibody				
Gt α rabbit DyLight 488	Abcam	ab96899	1:200	IF, 3-D cyst
Gt α mouse DyLight 488	Abcam	ab96879	1:200	IF
Gt α mouse DyLight 550	Abcam	ab96872	1:200	IF
Gt α rabbit DyLight 550	Abcam	ab96884	1:200	IF
Fluorescent dyes				
LysoTracker Red	Invitrogen	L7528	1:1,000	IF
Acridine Orange	Immunochemistry Technologies	6130	1:1,000	IF

3-D, three-dimensional; CST, Cell Signaling Technologies; FFPE, formalin-fixed parafin-embedded; IF, immunofluorescence; IHC, immunohistochemistry; MDCK, Madin-Darby canine kidney.

For tissue immunohistochemistry, FFPE sections (5 µm) were cleared in xylene (2 × 5 min) and rehydrated through a 100% (2 × 5 min), 95% (2 × 3 min), 80% (1 × 1 min), and 70% (1 × 1 min) alcohol series before being placed in distilled water. For heat-induced epitope retrieval, sections were placed in 10 mM citric acid (pH 6.0) and microwaved on full power for 3 × 5 min. Sections were allowed to cool in antigen retrieval solution for 20 min and subsequently placed in 1× Tris-buffered saline (TBS; pH 7.6). Sections were removed, and a hydrophobic barrier was drawn around each section, which were subsequently incubated with Peroxidazed-1 (Cat. No. PX968, Biocare Medical) for 5 min at room temperature. Sections were rinsed in 1× TBS and incubated with Background Sniper (Cat. No. BS966, Biocare Medical) for 10 min at room temperature. Sections were rinsed in 1× TBS + 0.05% Tween 20 (TBST) before incubation with primary antibody diluted in DaVinci Green Diluent (Cat. No. PD900, Biocare Medical) at the dilutions shown in Table 2. For negative controls, sections were incubated with polymer negative control serum (Cat. No. NC499, Biocare Medical), which all showed no evidence of staining. Sections were incubated with primary antibodies overnight at 4°C in a humidified chamber. The following day, sections were rinsed with 1× TBST, and MACH4 polymer (Cat. No. M4U534, Biocare Medical) was applied to each section and incubated for 30 min at room temperature. Sections were rinsed in 1× TBST followed by a rinse with 1× TBS. The binding of primary antibodies to the tissue sections was visualized with Deep Space Black chromogen (Cat. No. BRI4015, Biocare Medical), and color development was monitored under an Olympus CK 2 microscope. All sections incubated with the same antibody were incubated for the same length of time within each experiment. Sections were counterstained in either methyl green (Cat. No. H-3402, Vector Labs) for 5 min or nuclear fast red (Cat. No. H-3403, Vector Labs) for 30 s and run under tap water until they ran clear. Sections were dehydrated in 95% alcohol (2 × 5 dips) and 100% alcohol (2 × 10 dips), cleared in two changes of xylene (2 × 5 min), and permanently mounted with Permount (Cat. No. SP15, Fisher Scientific). For dual tissue immunofluorescence with TFEB and Hoechst 33342, tissue sections were treated similarly to above with the following modifications: insertion of a 20-min Sudan B black (Cat. No. 199664, Sigma-Aldrich, 0.1% in 70% ethanol) blocking step following the 70% ethanol step and before a distilled water wash and antigen retrieval were performed. Hoechst 33342 was added with the fluorescent-conjugated secondary antibody, and sections were incubated for 1 h at 37°C before mounting slides with Immu-mount (Cat. No. FIS9990412, Fisher Scientific). The images presented are representative of acquired images obtained from either three different animals (see Table 1) or four different human ADPKD samples (see Table 3) per antibody.

**Table 3.** Details of the human ADPKD and NHK samples used in this study

Identifier	Age, yr	Sex	Blood Urea Nitrogen	Creatine	Representative Images
ADPKD K472	52	Female	25	1.51	Fig. 10, *A*, C, and *E*; Supplemental Fig. S8, *A*–*C*
ADPKD K445	49	Male	17	1.26	Supplemental Figs. S7*A*, S9*A*, and S10*A*
ADPKD K447	52	Male	40	4.00	Supplemental Figs. S7*B*, S9*B*, and S10*B*
ADPKD K478	54	Male	30	5.75	Supplemental Figs. S7*C*, S9*C*, and S10*C*
NHK K465	55	Female			Fig. 10, *B, D*, and *F*; Supplemental Fig. S8, *D*–*F*
NHK K451	58	Male			Supplemental Figs. 7*D*, 9*D*, and 10*D*
NHK K453	53	Male			Supplemental Figs. S7*E*, S9*E*, and S10*E*
NHK K454	57	Male			Supplemental Figs. S7*F*, S9*F*, and S10*F*

ADPKD, autosomal dominant polycystic kidney disease; NHK, normal human kidney.

### Quantification of Tfeb Immunostaining

To semiquantify Tfeb immunohistochemistry signals, pathology analysis software (QuPath, version 0.3.2) was used. In brief, immunohistochemical images were opened, and cysts (red), tubules (blue), and glomeruli (orange) were manually annotated and classified using the polygon selection tool. A cyst was defined as a cellular structure having a distinct white background within the luminal space. For the identification of positive and negative cells, the optical density sum image, which is agnostic to the color of the stain and instead uses the sum of the brightness intensity within the image, was used. Cellular analysis parameters were adjusted and optimized to enable the separation and segregation of the cytoplasm and nucleus. Intraluminal, noncellular, and nonspecific detections were manually removed. To enable specific relative quantification of the nuclear signal, the image was subjected to intensity thresholding based on the nuclear optical density mean value and assigned a binary positive or negative score. The relative nuclear enrichment percentage was calculated as the number of positive cells expressed as a percentage of the total number of cells within the compartments classified as cyst or noncyst.

### Particle Determination and Analysis

Images of LysoTracker red uptake and Lamtor4 immunocytochemistry were processed for particle analysis in FIJI as follows. Images were altered to 16-bit and then 8-bit and subjected to adjustment using the auto local thresholding feature (Phansalkar method, radius of 15). Images were subjected to the watershed filer to separate merged particles before using the analyze particles feature with the pixel unit box unchecked and the size set to 20 – infinity to determine the number of particles per image, mean fluorescence intensity, and average particle size. Since there was significant variation in the intensity of the nuclear staining between individual cells, the total number of cells was calculated manually using the multipoint counting feature. Five individual fields were analyzed with a minimum of 200 total cells in each field, and representative images of individual postprocessed cells are shown in the respective figures.

### Real-Time Quantitative PCR

Total RNA was isolated from *Pkd1* KO and *Pkd1* wild-type MEFs grown to ∼80% confluence in T75 flasks with the RNeasy Plus Mini Kit (Cat. No. 74034, Qiagen) according to the manufacturer’s protocol. Following total RNA isolation, RNA was quantified, and the integrity was determined (Bioanalyzer 2100, Agilent). A total of 1 µg of total RNA was used to generate cDNA using the SuperScript IV first-strand synthesis system (Cat. No. 18091050, Invitrogen) using a mixture of random hexamers and oligo dT primers. For each quantitative PCR, 0.4-µL cDNA was added to Power SYBR Green Master Mix (Cat. No. 4368577, Applied Biosystems) with gene-specific primers (IDT), and reactions were run on a quantitative PCR instrument (StepOne Real-Time PCR System, Applied Biosystems). Relative gene expression was normalized to two control genes (*Pol2ra* and *Ppia*), and results were normalized to the geometric mean. Experiments were carried out in triplicate.

### Microscopy and Processing of Acquired Images

Immunostained kidney sections were visualized with a Leica DM IRB microscope under bright-field imaging, and images were acquired with an Olympus DP70 camera via Olympus DP Manager software. All images were identically adjusted in GNU Image Manipulation Program (version2.10.32) to improve background and overall image clarity postacquisition; for cyst cultures, cyst diameters were calculated using the measure function. For two-dimensional immunocytochemistry experiments, images were acquired using the filter set appropriate for the fluorescence wavelength needed. For 3-D cyst cultures, images were acquired under the appropriate wavelength settings with a Leica SP8 confocal microscope using LAS-X software (Leica Microsystems). For postacquisition image analysis, two-dimensional cross-sections were generated in LAS-X, and representative 3-D cyst images were identically postprocessed in Imaris.

### Statistical Analyses

Data were analyzed by a Student’s *t* test or one-way ANOVA multiple comparison with a Tukey’s post hoc test (GraphPad Prism) depending on the number of samples being analyzed in each comparison. *P* values of <0.05 were considered significant.

## RESULTS

### Nuclear Tfeb Translocation in Models of Renal Cystic Disease

**Figure 1.**
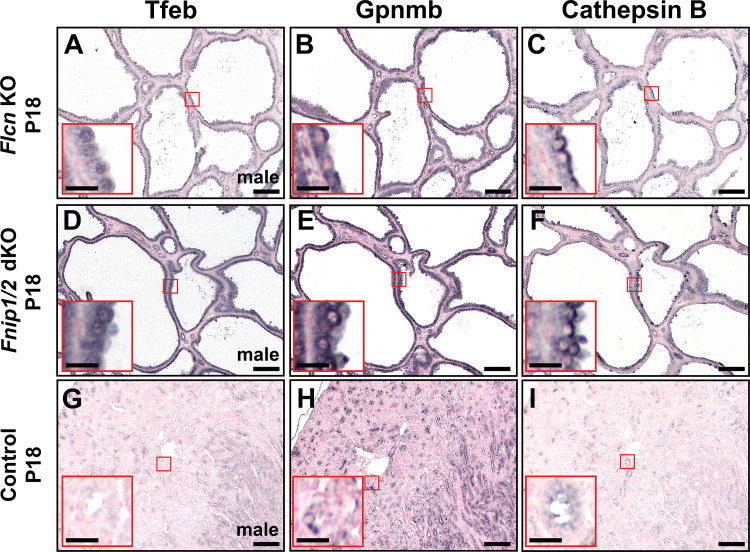
Transcription factor EB (Tfeb) upregulation and nuclear translocation in renal epithelial cells in the folliculin (*Flcn*) knockout (KO) and folliculin-interacting protein 1 and 2 (*Fnip1/2*) double KO (dKO) models of cystic disease. Immunohistochemistry was performed on serial renal formalin-fixed paraffin-embedded sections derived from *Flcn^fl/fl^:Ksp-Cre* (*Flcn* KO; *A–C*), *Fnipi1^fl/fl^:Fnip2^fl/fl^:Ksp-Cre* (*Fnip1/2* dKO; *D−F*) and aged-matched control (*Flcn^fl/+^:Fnip1^fl/+:^Fnip2^fl/+^:Ksp-Cre*; *G–I*) animals using antibodies raised against Tfeb (*A*, *D*, and *G*), glycoprotein nonmetastatic melanoma protein B (Gpnmb; *B*, *E*, and *H*), or cathepsin B (*C*, *F*, and *I*). A striking upregulation and nuclear translocation of Tfeb (*A* and *D*) were observed in renal cystic epithelial cells in kidneys from *Flcn* KO (*A*) and *Fnip1/2* dKO (*D*) animals compared with the low-level cytoplasmic staining of Tfeb observed in a subset of renal tubules from control kidneys (*G*). This nuclear translocation of Tfeb in renal cystic epithelial cells corresponded with a significant upregulation of Gpnmb (*B* and *E*), a known downstream target of Tfeb, which exhibited strong apical membrane staining compared with control renal tubules (*H*). A similar staining pattern was observed for the lysosomal-specific enzyme cathepsin B, another Tfeb target, which was significantly upregulated (*C* and *F*) and exhibited a strong apical localization pattern compared with the low expression evident in control renal tubules (*I*). Scale bars = 100 µm; scale bars in *insets* = 20 µm. Images are representative of five ×10 images acquired from three separate animals for each genotype. P18, *postnatal day 18*.

The expression and subcellular localization of Tfeb were first determined in multiple renal cystic disease models using immunohistochemistry. *Flcn^fl/fl^:Ksp-Cre* mice, a model of BHD syndrome, are based on kidney-specific deletion of *Flcn* (8). Tfeb staining revealed exclusive nuclear staining in most renal cystic epithelia (Fig. 1*A*). In contrast, predominantly apical membrane and cytoplasmic staining was observed in a subset of renal tubules from age-matched *Flcn^fl/+^:Ksp-Cre* kidneys (Fig. 1*G*). To further confirm nuclear localization, samples were subjected to dual immunofluorescence staining with Tfeb and the nuclear marker Hoechst 33342 (Supplemental Fig. S1). Renal cystic epithelial cells showed both upregulation and colocalization with Hoechst 33342 compared with the very low level of nuclear Tfeb observed in surrounding noncystic epithelial cells (Supplemental Fig. S1, *A–C*) and control samples (Supplemental Fig. S1, *D–F*).

A second model of renal cystic disease, *Fnip1/2^fl/fl^:Ksp-Cre* double KO, is based on deletion of *Fnip1* and *Fnip2* (8). Nuclear Tfeb in renal cysts and precystic lesions (Fig. 1*D*) was observed compared with heterozygous *Fnip1/2^fl/+^*:*Ksp-Cre* control samples (Fig. 1*G*). Thus, Tfeb nuclear translocation is associated with deletion of either *Flcn* or of its interacting partners, *Fnip1* and *Fnip2*.

Tfeb is known to induce coordinated lysosomal expression and regulation of (CLEAR) network genes that are involved in autophagy and lysosomal biogenesis (5, 11). To assess the functionality of Tfeb translocation, two Tfeb-responsive gene products were first studied, Gpnmb (12) and CTSB. Nuclear staining of Tfeb in cystic epithelial cells was associated with a significant upregulation of Gpmnb (Fig. 1, *B* and *E*) in serial sections of renal tissue from both KO models. Strong apical membrane staining of Gpmnb was seen in cystic compared with control renal tubules (Fig. 1*H*). A similar apical staining pattern was observed for CTSB, which was significantly upregulated (Fig. 1, *C* and *F*) compared with the low expression evident in control tubules (Fig. 1*I*).

### Nuclear Translocation of Tfeb and Tfe3 and Their Association With mTORC1 Activity

**Figure 2.**
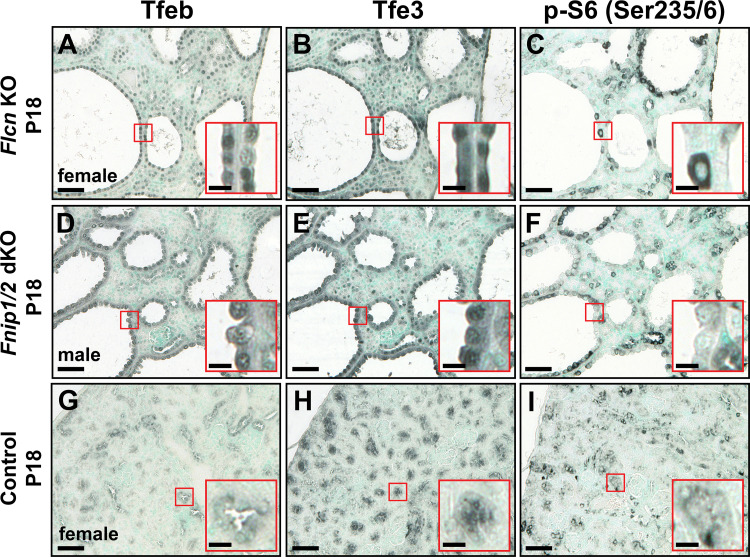
Cyst-specific nuclear translocation of transcription factor EB (Tfeb) and transcription factor E3 (Tfe3) and the activation status of mammalian target of rapamycin complex 1 (mTORC1). Serial renal formalin-fixed paraffin-embedded sections derived from folliculin (*Flcn*)*^fl/fl^:Ksp-Cre* [*Flcn* knockout (KO); *A–C*], folliculin-interacting protein (*Fnip*)*1^fl/fl^:Fnip2^fl/fl^Ksp-Cre* [*Fnip1/2* double KO (dKO); *D–F*], or control (*G–I*) animals were subjected to immunohistochemistry using antibodies raised against Tfeb (*A*, *D*, and *G*), Tfe3 (*B*, *E*, and *H*), or phosphorylated (p)-S6 (Ser^235/236^; *C*, *F*, and *I*). Nuclear Tfe3 was detected in most cystic epithelial cells in both models (*B* and *E*) compared with control (*H*) renal epithelia, which showed no evidence of nuclear translocation. Activation of mTORC1, as assessed by p-S6 (Ser^235/236^; *C* and *F*), displayed a sporadic and patchy staining pattern in tissue sections from cystic kidneys in which nuclear Tfeb and Tfe3 staining was predominant. Scale bars = 50 µm; scale bars in *insets* = 10 µm. Images are representative of five ×20 images acquired from three separate animals for each genotype. P18, *postnatal day 18*.

In addition to the presence of nuclear Tfeb in renal cysts (Fig. 2, *A* and *D*), a similar and pronounced nuclear translocation of Tfe3, another melanocyte-inducing transcription family member, was observed in cyst epithelia in both *Flcn^fl/fl^:Ksp-Cre* (Fig. 2*B*) and *Fnip1/2^fl/fl^:Ksp-Cre* double KO (Fig. 2*E*) models compared with control tubular epithelia (Fig. 2, *G* and *H*). mTORC1 is known to control the activity of Tfeb via phosphorylation at the lysosome and cytosolic sequestration (13). mTORC1 activation was probed by determining in situ expression of phospho-S6 (Ser^235/236^), a surrogate marker of mTORC1 activity (14). mTORC1 activation was only observed in a subset of individual cells within single cysts (Fig. 2, *C* and *F*), consistent with a limited correlation between mTOR activity and Tfeb/3 activation.

### Age-Dependent Changes in Nuclear Tfeb Translocation

**Figure 3.**
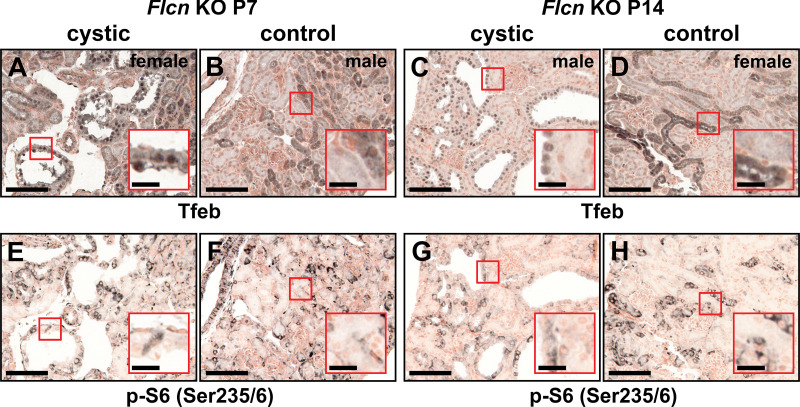
Age-associated nuclear translocation of transcription factor EB (Tfeb) in renal cysts early in the development of cystic disease. Serial renal formalin-fixed paraffin-embedded sections derived from folliculin (*Flcn*)*^fl/fl^:Ksp-Cre* [*Flcn* knockout (KO); *A* and *E*] or control (*Flcn^fl/+^:Ksp-Cre*) animals (*B* and *F*) at *postnatal day 7* (P7) were subjected to immunohistochemistry using an antibody against Tfeb or phosphorylated (p-)S6 (Ser^235/236^), respectively. All renal cysts stained positively for nuclear Tfeb (*A*) with very few nuclear-positive epithelial cells detected in control samples (*B*). Activation of mammalian target of rapamycin complex 1 was only observed in a subset of cystic epithelial cells (*E*) and was similar to the staining pattern observed in control samples (*F*). At *postnatal day 14* (P14), almost all cystic renal epithelial cells were positive for nuclear Tfeb (*C*) compared with normal renal tubules in control animals (*D*), in which the Tfeb signal was mostly cytoplasmic. p-S6 (Ser^235/236^) staining was sporadic in renal cysts (*G*) and mimicked the staining pattern observed in control samples (*H*). Scale bars = 100 µm; scale bars in *insets* = 20 µm. Images are representative of five ×20 images acquired from three separate animals for each genotype.

*Flcn^fl/fl^:Ksp-Cre* KO mouse kidneys from *postnatal day 7* and *14* mice were stained for Tfeb and phospho-S6 (Ser^235/236^) to assess age and cyst development. Nuclear Tfeb was evident at *postnatal days 7* and *14* in KO cystic but not control epithelia (Fig. 3, *A* and *C*). In contrast, control age-matched kidney samples exhibited Tfeb nuclear translocation in only a few renal tubules (Fig. 3, *B* and *D*). To further determine upregulation and nuclear translocation of Tfeb, coimmunostaining of Tfeb with the nuclear marker Hoechst 33342 (Supplemental Figs. S2 and S3) was performed. At both time points, a significant upregulation and pronounced nuclear translocation of Tfeb (Supplemental Figs. S2, *A–C*, and S3, *A–C*) were observed compared with the low levels of Tfeb observed in surrounding normal epithelial cells and control samples (Supplemental Figs. S2, *D–F*, and S3, *D–F*).

To quantify the observed Tfeb nuclear translocation, the pathology software program QuPath (see materials and methods), was used to examine the nuclear Tfeb immunohistochemistry signals in cystic versus noncystic epithelial cell compartments (Supplemental Fig. S4). Using this approach and outlined methodology, nuclear Tfeb was present in 87.89% of cystic cells compared with only 20.00% of noncystic cells (see Supplemental Fig. S4*F* for quantification). Taken together, these data demonstrate that there is a significantly nuclear enrichment of Tfeb in renal cystic cells.

Like that observed in *postnatal day 18* samples, there was no apparent correlation between mTORC1 activity and Tfeb nuclear localization (Fig. 3, *E–H*) since Tfeb was observed in the nucleus in the vast majority of cystic epithelial cells, whereas phospho-S6 (Ser^235/236^) staining, a surrogate maker of mTORC1 activity, was only observed in a subset of cystic cells (see Fig. 3, *insets*). These results suggest that there is a disconnect between Tfeb and mTORC1 in renal cystic cells and that other pathway or pathways might be responsible for cyst-specific Tfeb activation.

### Nuclear Tfeb Translocation in *Pkd1* KO Mice

**Figure 4.**
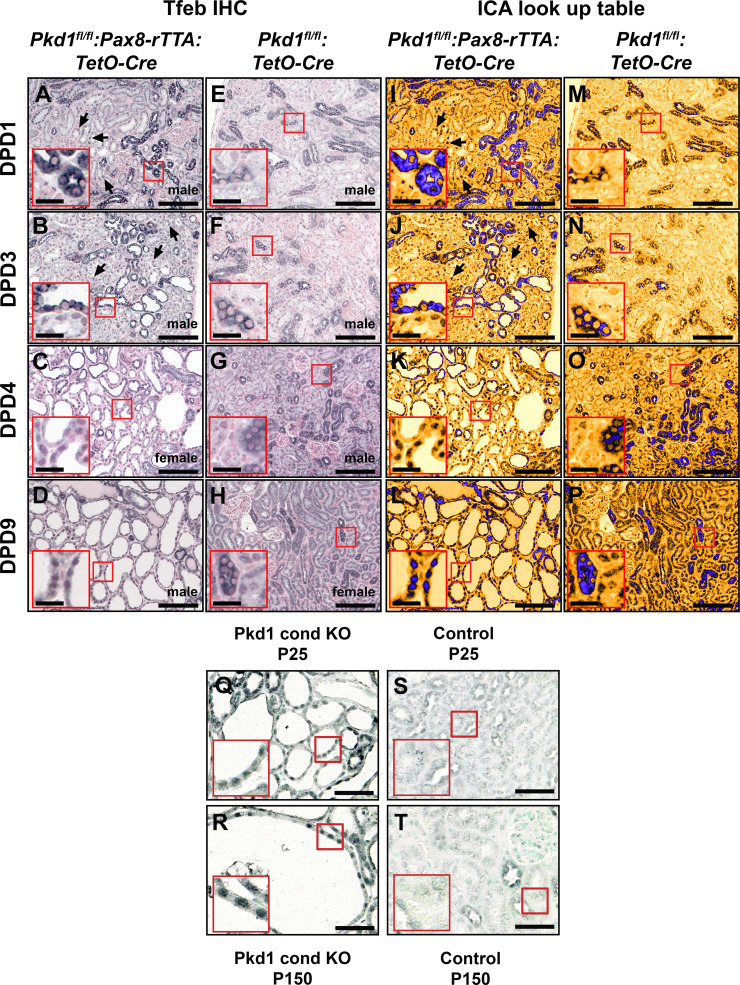
Transcription factor EB (Tfeb) upregulation and nuclear translocation following deletion of the polycystin-1 (*Pkd1*) gene. *A−H*: formalin-fixed paraffin-embedded renal sections derived from *Pkd1^fl/fl^:Pax8-rTTA:TetO-Cre* (*Pkd1* iKO) mice that had been induced with doxycycline (DOX) at *postnatal days 10* and *11* and isolated 1 day (*A*; DPD1), 3 days (*B*; DPD3), 4 days (*C*; DPD4), and 9 days (*D*; DPD9) post-DOX were subjected to immunostaining with a Tfeb antibody and compared with age-matched control (DOX-induced *Pkd1^fl/fl^:TetO-Cre* without Pax8-rTTA) animals (*E–H*). Nuclear translocation of Tfeb was observed in renal epithelial cells as early as DPD1 (*A*, black arrows), and the overall frequency of nuclear Tfeb translocation increased with age compared with control samples. Significant cytoplasmic punctate accumulation of Tfeb was evident at the DPD1 (*A*) and DPD3 (*B*) time points compared with control samples (*E* and *F*, respectively). To improve visual contrast, color images were converted to 8-bit grayscale, and a lookup table (ICA) was used to represent the relative intensity of the pixel data (*I–P*). Scale bars = 100 µm; scale bars in *insets* = 20 µm. Images are representative of five ×40 images acquired from three separate animals for each genotype. *Q*−*T*: Tfeb staining was performed on renal sections derived from *Pkd1* iKO mice harvested at *day 25* (*Q*) or *day 150* (*R*). There was a pronounced upregulation and nuclear translocation in renal cystic epithelial cells from mice that were 2 or 20 wk postinduction. In contrast, age-matched control samples (*S* and *T*) showed very low levels of Tfeb expression in normal renal tubules as well as little evidence of nuclear translocation at these two time points. Scale bars = 100 µm; scale bars in *insets* = 20 µm. Images are representative of five ×40 images acquired from three separate animals for each genotype.

A renal-specific, DOX-inducible *Pkd1* KO model (*Pkd1^fl/fl^:Pax8-rTTA:TetO-Cre*) was next used to determine if Tfeb was similarly affected in renal cysts that arise due to *Pkd1* deletion. The age-dependent expression and subcellular localization of Tfeb were evaluated in *Pkd1^fl/fl^:Pax8-rTTA:TetO-Cre* KO mice in which *Pkd1* deletion was induced at *days 10* and *11* postpartum. Tfeb staining was performed in renal sections isolated 1 day (Fig. 4*A*), 3 days (Fig. 4*B*), 4 days (Fig. 4*C*), and 9 days (Fig. 4*D*) post-DOX and compared with age-matched controls, DOX-induced *Pkd1^fl/fl^:TetO-Cre* without Pax8-rTTA (Fig. 4, *E–H*). Nuclear staining of Tfeb in KO tubules was significantly increased relative to control tubules at 4 and 9 days post-DOX. Nuclear translocation of Tfeb was observed before cyst formation in some cells at 1 day post-DOX from *Pkd1* KO kidneys. The increase in nuclear Tfeb staining in *Pkd1* KO kidneys (Fig. 4, *I*–*L*) relative to control kidneys (Fig. 4, *M*–*P*) was highlighted by application of a look-up table (ICA). Tfeb upregulation and nuclear translocation were further confirmed by costaining with the nuclear marker Hoechst 33342 (Supplemental Fig. S5), which revealed colocalization in renal cystic epithelial cells (Supplemental Fig. S5, *A–C*) and low-level staining in control epithelial cells (Supplemental Fig. S5, *D–F*) in *postnatal day 20* (9 days post-DOX) samples.

Additional analysis of samples isolated at *postnatal day 25* (14 days post-DOX) revealed nuclear enrichment of Tfeb in renal cystic epithelial cells (Fig. 4*Q*) compared with normal tubules (Fig. 4*S*). The pattern of cystic epithelial cell Tfeb nuclear translocation appeared to persist as it was also observed in cystic samples at *postnatal day* 150 following DOX administration at *postnatal days 27−29* (Fig. 4, *R* vs. *T*). Thus, nuclear translocation of Tfeb appears to be both an early and a sustained event in the development of renal cysts in both *Flcn^fl/fl^:Ksp-Cre* and *Pkd1^fl/fl^:Pax8-rTTA:TetO-Cre* models.

### Tfeb in MEFs Lacking *Pkd1*

**Figure 5.**
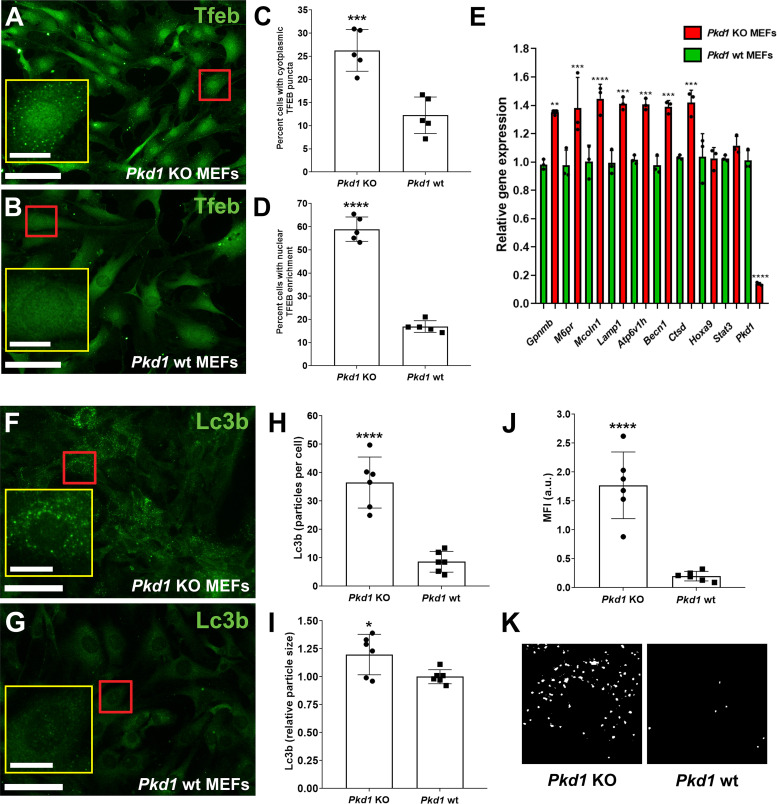
Effect of deletion of polycystin-1 (*Pkd1*) on nuclear enrichment of transcription factor EB (Tfeb) and light chain 3B (Lc3b) in mouse embryonic fibroblasts (MEFs). *Pkd1* knockout (KO) and *Pkd1* wild-type (wt) MEFs were subjected to immunocytochemistry using a Tfeb antibody. *Pkd1* KO MEFs (*A*) exhibited evidence of significant nuclear enrichment and the appearance of cytoplasmic puncta compared with *Pkd1* wt MEFs (*B*). Scale bars = 100 µm; scale bars in *insets* = 20 µm. Images are representative of five ×20 images acquired from three independent experiments and a total *n* > 500 cells. Quantification of cytoplasmic puncta (*C*) and nuclear TFEB enrichment (*D*) revealed significant (****P < 0.0001 in *C* and ***P < 0.001 in *D*) upregulation in *Pkd1* KO MEFs compared with *Pkd1* wt MEFs. *E*: quantitative PCR analysis of previously identified Tfeb-associated and nonassociated target genes (*Hoxa9* and *Stat3*) in *Pkd1* KO MEFs compared with *Pkd1* wt MEFs. As expected, a significant reduction in *Pkd1* expression was observed in *Pkd1* KO vs. wt MEFs. **P < 0.01, ***P < 0.001, and ****P < 0.0001. *Pkd1* KO (*F*) and *Pkd1* wild-type (*G*) MEFs were subjected to immunostaining using an antibody raised against Lc3b. Compared with *Pkd1* wt MEFs, *Pkd1* KO MEFs exhibited a significant increase in Lc3b-positive cytoplasmic puncta. Quantification of representative acquired images revealed a significant increase in the number of Lc3b particles per cell (*H*; ****P < 0.0001), relative particle size (*I*; *P < 0.05), and mean fluorescence intensity [MFI, in arbitrary units (au); *J*, ****P < 0.0001) in *Pkd1* KO MEFs compared with *Pkd1* wt MEFs. *K*: representative examples of the results of the image processing procedure used for particle analysis, which is described in detail in materials and methods. Scale bars = 100 µm; scale bars in *insets* = 20 µm. Images are representative of six ×20 images acquired from three independent experiments and a total *n* > 500 cells.

*Pkd1* KO and *Pkd1* wild-type MEFs isolated from *embryonic day 12.5* animals were used as an in vitro cellular model system of *Pkd1* gene deletion to further probe functional downstream correlates of nuclear Tfeb translocation (Fig. 5). Like that observed in cystic renal epithelial cells in the *Pkd1^fl/fl^:Pax8-rTTA:TetO-Cre* model, *Pkd1* KO MEFs (Fig. 5, *A* and *D*) showed significant nuclear enrichment of Tfeb compared with *Pkd1* wild-type MEFs (Fig. 5, *B* and *D*). *Pkd1* KO MEF cells also exhibited bright cytoplasmic Tfeb-positive puncta compared with wild-type MEFs (Fig. 5, *A* and *C* vs. *B* and *C*). As observed with the other murine models of renal cystic disease examined, costaining of Tfeb with the nuclear marker Hoechst 33342 as assessed by immunofluorescence (see Supplemental Fig. S5) revealed upregulation and significant nuclear colocalization (Supplemental Fig. S5, *A–C*) compared with wild-type cells (Supplemental Fig. S5, *D–F*).

To determine whether the observed nuclear translocation of Tfeb in *Pkd1* KO MEFs was correlated with the induction of previously defined Tfeb targets such as those involved in lysosomal biogenesis (11) and autophagy (15), a subset of genes was examined by real-time quantitative PCR (Fig. 5*E*). An increase in several known Tfeb targets was observed in *Pkd1* KO compared with *Pkd1* wild-type MEFs. These included transcripts associated with lysosomal biogenesis [mucolipin transient receptor potential (TRP) cation channel 1 (*Mcoln1*), lysosomal-associated membrane protein 1 (*Lamp1*), *Ctsd*, and *Atpv1h*], autophagy [beclin 1 (*Becn1*)], and lysosomal protein sorting [mannose-6-phosphate receptor, cation dependent (*M6pr*)]. In contrast, homeobox A9 (Hoxa9) and signal transducer and activator of transcription 3 (Stat3) were not differentially expressed between *Pkd1* KO and wild-type MEFs. Gpnmb (osteoactivin), a protein under Tfeb regulation normally associated with cancer metastasis and neuroinflammation, was also increased in *Pkd1* KO MEFs. Thus, nuclear Tfeb is transcriptionally active in the absence of *Pkd1*.

### *Pkd1* Deletion Results in Changes in Autophagy

Tfeb has been demonstrated to be a master regulator of the autophagic pathway (16). Therefore, to determine whether deletion of *Pkd1* and the subsequent activation of Tfeb is associated with changes in autophagy, the expression and subcellular localization of Lc3b, an established marker of autophagy (17), were examined. A marked increase in bright Lc3b-positive cytoplasmic puncta was observed in *Pkd1* KO compared with *Pkd1* wild-type MEFs, which exhibited a general diffuse cytoplasmic pattern of Lc3b staining (Fig. 5, *F* and *G*). The increase in Lc3b in *Pkd1* KO MEFs was further confirmed by increases in particles per cell, particle size, and mean fluorescence intensity (Fig. 5, *H*–*K*).

**Figure 6.**
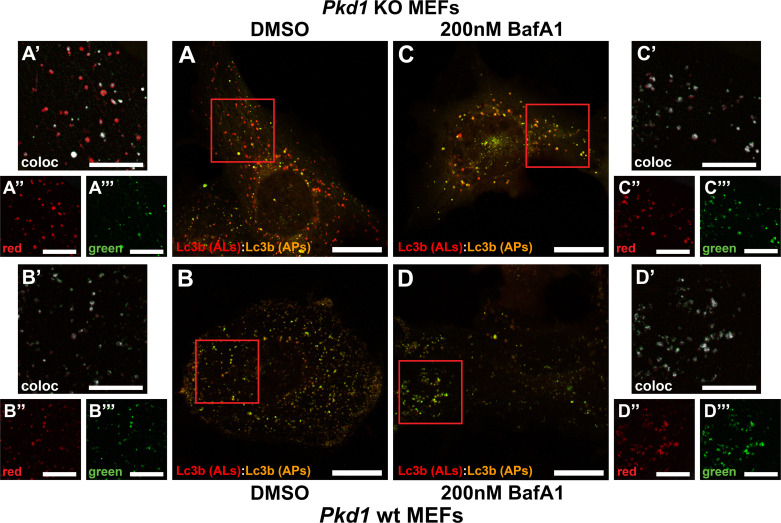
Autophagic flux as assessed by a dual red fluorescent protein (RFP)-green fluorescent protein (GFP)-light chain 3B (Lc3b) reporter. Polycystin-1 (*Pkd1*) knockout (KO; *A* and *C*) and *Pkd1* wild-type (wt; *B* and *D*) mouse embryonic fibroblasts (MEFs) were transduced with a dual RFP-GFP-Lc3b reporter 24 h before live cell imaging analysis. *Pkd1* KO MEFs (*A*) exhibited an increase in predominantly red puncta indicative of autolysosomes (ALs) compared with *Pkd1* wt MEFs (*B*). In contrast, incubation of *Pkd1* KO (*C*) or *Pkd1* wt (*D*) MEFs with 200 nM brefeldin A1 (BafA1) resulted in predominantly dual red and green autophagosomes (APs) indicative of a similar block in autophagosome-lysosome fusion. *Inset* zooms represent colocalized pixels in white (*A′– D′*) and individual red (*A′′–D′′*) and green (*A′′′–D′′′*) channels obtained following background subtraction. Scale bars = 20 µm; scale bars in *insets* = 10 µm. Images are representative of three independent experiments in which 20 transduced cells were analyzed per experiment.

To explore this observation further, autophagic flux was measured in a more detailed way by transducing *Pkd1* KO and wild-type MEFs with a dual-color RFP-GFP-Lc3b reporter construct. Using this construct, autophagosomes express both red and green. In contrast, when autophagosomes fuse with lysosomes to form autolysosomes, the green signal is quenched in the resulting acidic environment and autolysosomes appear predominantly red (18). Using this approach, a significant increase in predominantly red particles in transduced *Pkd1* KO cells (Fig. 6*A*) was observed compared with *Pkd1* wild-type cells (Fig. 6*B*), indicative of an increase in autolysosomes and autophagic flux. Following incubation of cells with 200 nM brefeldin A1, a V-ATPase inhibitor that blocks the fusion of autophagosomes with lysosomes, both *Pkd1* KO (Fig. 6*C*) and wild-type (Fig. 6*D*) MEFs accumulated intracellular vesicles that were both red and green, indicative of autophagosomes and a similar block in autophagic flux.

**Figure 7.**
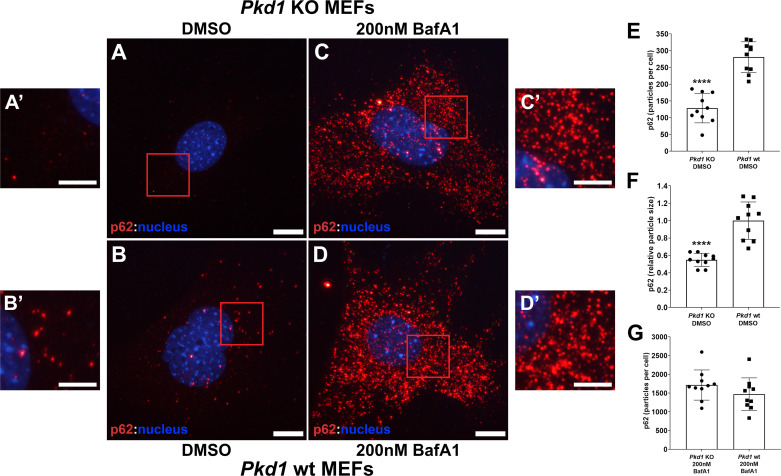
Analysis of p62 cytoplasmic puncta. Polycystin-1 (*Pkd1*) knockout ((KO; *A* and *C*, insets *A*’ and *C*’) and *Pkd1* wild-type (wt; *B* and *D*, insets *B*’ and *D*’) mouse embryonic fibroblasts (MEFs) were subjected to immunostaining using an antibody raised against p62. *Pkd1* KO MEFs (*A*) exhibited a significant reduction in the number (*E*; ****P < 0.0001) and relative size (*F*; ****P < 0.0001) of cytoplasmic p62 puncta compared with *Pkd1* wt MEFs (*B*). Treatment of cells with 200 nM brefeldin A1 (BafA1) resulted in a significant increase in the number (*G*) of p62 puncta that was similar between *Pkd1* KO (*C*) and *Pkd1* wt (*D*) MEFs consistent with a block in autophagosome-lysosome fusion and subsequent accumulation of nondegraded p62 protein. Scale bars = 20 µm; scale bars in *insets* = 10 µm. Images are representative of five independent experiments in which 10 cells were analyzed per experiment.

An increase in autophagic flux and the subsequent generation of autolysosomes should result in a decrease in SQSTM1/p62 due to its increased degradation (18). Immunostaining fixed MEFs with an antibody raised against p62 revealed a significant reduction in the number (quantified in Fig. 7*E*) and increase in size (quantified in Fig. 7*F*) of intracellular p62 puncta in *Pkd1* KO MEFs (Fig. 7, *A* and *A**′*) compared with *Pkd1* wild-type MEFs (Fig. 7, *B* and *B**′*). In contrast, when the fusion of autophagosomes and lysosomes was blocked with 200 nM brefeldin A1, p62-positive puncta greatly accumulated due to its inability to be degraded and were similar in *Pkd1* KO and wild-type MEFs (Figs. 7, *C* and *C’* and 7, *D* and *D'*, quantified in *G*) (19). Taken together, these findings are consistent with Tfeb stimulated autophagy, which is increased in the absence of *Pkd1* expression.

### Changes in Lysosome Size and Distribution in the Absence of *Pkd1*

**Figure 8.**
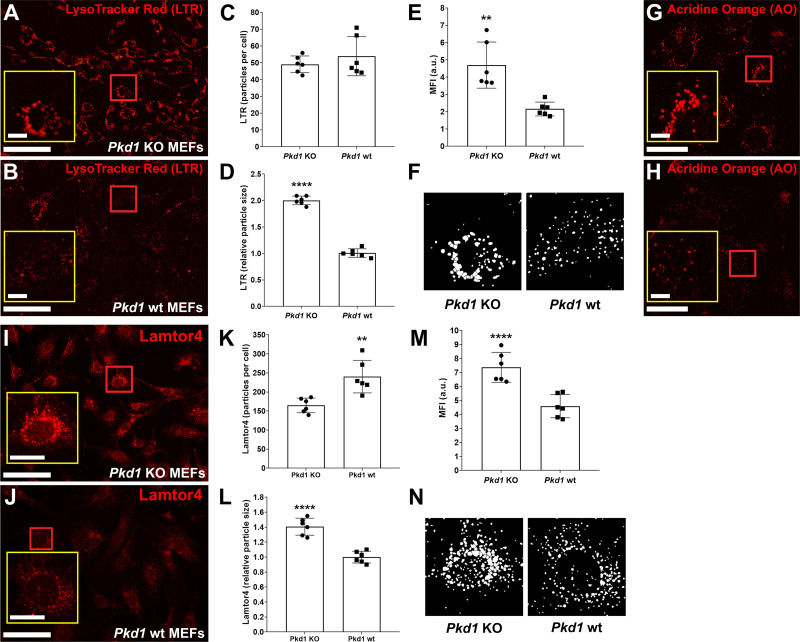
Intracellular distribution of LysoTracker red (LTR), acridine orange (AO), and late endosomal/lysosomal adaptor and MAPK and mammalian target of rapamycin activator 4 (Lamtor4) in polycystin-1 (*Pkd1*) KO and wt mouse embryonic fibroblasts (MEFs). *Pkd1* KO (*A*) and *Pkd1* wt (*B*) MEFs were incubated with LTR for 30 min before live-cell analysis. *Pkd1* KO MEFs exhibited condensed perinuclear accumulation of LTR (*A*) compared with *Pkd1* wt MEFs (*B*), in which these signals were more evenly distributed throughout the cytoplasm. Although quantification of representative acquired images revealed no difference in the number of LTR particles per cell (*C*), a significant increase in relative particle size (*D*; ****P < 0.0001) and mean fluorescence intensity [MFI, in arbitrary units (au); *E*; **P < 0.01] was observed in *Pkd1* KO MEFs compared with *Pkd1* wt MEFs. *F*: representative example of the results of the image processing procedure used for particle analysis, which is described in detail in materials and methods. Comparable results were obtained using AO, which exhibited a clustered perinuclear distribution in *Pkd1* KO MEFs (*G*) compared with a more diffuse distribution in *Pkd1* wt MEFs (*H*). Scale bars = 100 µm in *A* and *B* and 50 µm in *G* and *H*; scale bars in *insets* = 10 µm. Images are representative of six ×20 images acquired from three independent experiments and a total *n* > 500 cells. *Pkd1* KO (*I*) and *Pkd1* wt MEFs (*J*) were fixed and subjected to immunostaining with an antibody raised against Lamtor4. Quantification of representative acquired images revealed a significant decrease in the number of Lamtor4-positive particles (*K*; **P < 0.01) and an increase in relative particle size (*L*; ****P < 0.0001) and MFI (*M*; ****P < 0.0001) in *Pkd1* KO MEFs compared with *Pkd1* wt MEFs. *N*: representative example of the results of the image processing procedure used for particle analysis, which is described in detail in materials and methods. Scale bars = 100 µm; scale bars in *insets* = 20 µm. Images are representative of six ×20 images acquired from three independent experiments and a total *n* > 500 cells.

Tfeb-mediated lysosomal biogenesis was studied with LysoTracker red uptake in *Pkd1* KO and *Pkd1* wild-type MEFs (Fig. 8). *Pkd1* KO MEFs exhibited increased intracellular vesicular uptake of LysoTracker red compared with *Pkd1* wild-type cells based on particle size and intensity (Fig. 8, *A*–*E*). Pronounced perinuclear accumulation of LysoTracker red-positive puncta in *Pkd1* KO MEFs was evident compared with the dispersed and diffuse cytoplasmic staining observed in *Pkd1* wild-type MEFs (Fig. 8, *B* and *F*). These changes were most evident as an increase in particle size (Fig. 8*D*) and mean fluorescence intensity (Fig. 8*E*) and not in discernable particle number (Fig. 8*C*).

Acridine orange, a dye that accumulates in acidic vesicular organelles such as lysosomes, where it undergoes a metachromatic shift from green to red, was used. Using this approach, a pronounced perinuclear accumulation of acridine orange in the red channel in *Pkd1* KO MEFs (Fig. 8*G*) compared to wild-type MEFs (Fig. 8*H*), in which the acridine orange signal was more broadly distributed in the cytoplasmic compartment, was confirmed. These results support those obtained with LysoTracker red and further demonstrate that the lysosomal compartment undergoes repositioning in the absence of *Pkd1*.

Since the uptake of lysosomotropic dyes such as LysoTracker red and acridine orange might be altered by lysosomal pH, which may be changed following Tfeb activation, staining of Lamtor4, a protein that colocalizes with lysosomal markers such as Arl8b, lyspersin, and Lamp1 (20), was performed to confirm these observed changes (Fig. 8, *I*–*N*). Like LysoTracker red and acridine orange uptake, an increase in the perinuclear distribution of Lamtor4 in *Pkd1* KO MEFs was observed compared with *Pkd1* wild-type MEFs (Fig. 8, *I* vs. *J*). Quantification revealed a significant increase in relative Lamtor4-positive particle size (Fig. 8*L*) and mean fluorescence intensity (Fig. 8*M*) correlating with a decrease in Lamtor4-positive particle number (Fig. 8*K*). These data provide experimental evidence that deletion of *Pkd1* and subsequent activation of Tfeb results in a lysosomal positioning defect.

### Tfeb Nuclear Translocation and Cyst Size in MDCK Cells

**Figure 9.**
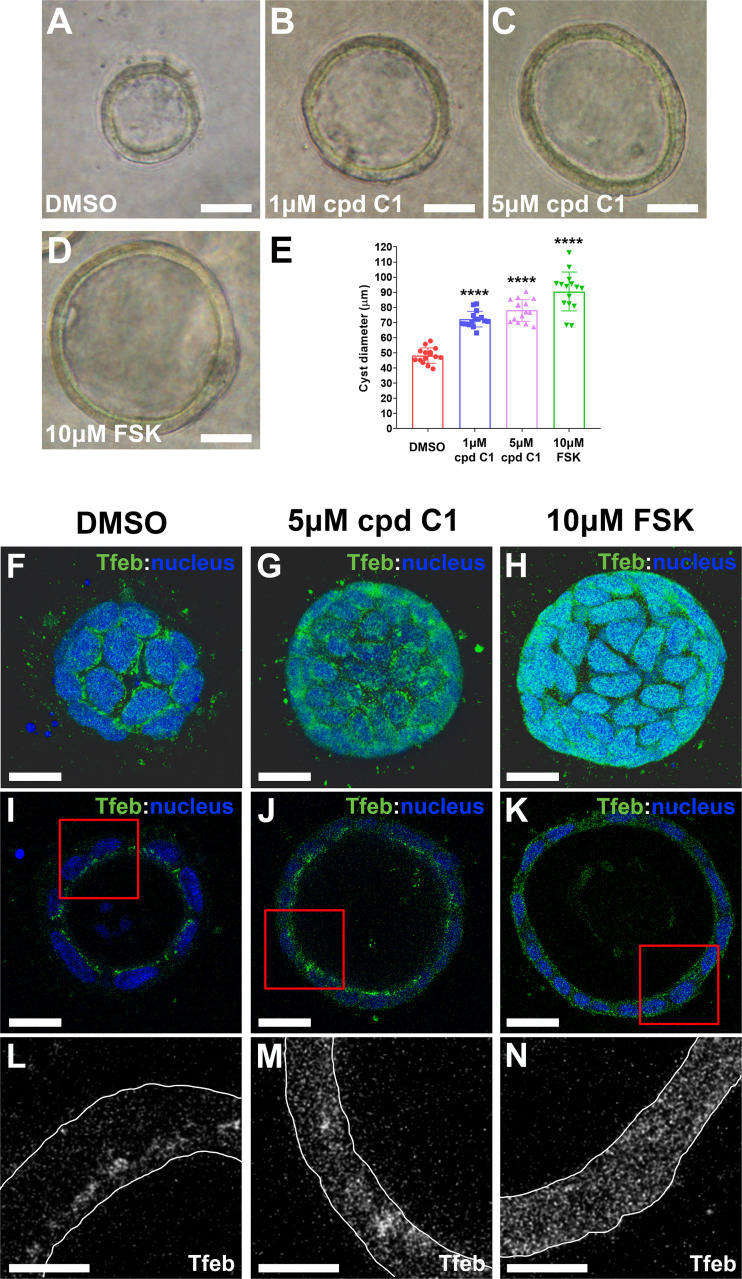
Effect of pharmacological transcription factor EB (TFEB) activation on cyst size in an in vitro three-dimensional cell culture model. Madin-Darby canine kidney cells were grown in a collagen (3 mg/mL) matrix in 15-well µ-slides for 5 days before incubation with 1 µM compound C1 (cpd C1; *B*), 5 µM compound C1 (*C*), or 10 µM forskolin (FSK; *D*) daily for 4 days before the acquisition of phase-contrast images on *day 9*. *E*: quantification of cyst diameters revealed a significant increase in cyst diameter in compound C1-treated (*B* and *C*) and FSK-treated positive control (*D*) cyst cultures compared with the DMSO-treated negative control condition (*A*). Scale bars = 25 µm; images are representative of 15 cysts acquired from each of 3 independent experiments. *F−N*: immunostaining of cyst cultures for TFEB represented in three-dimensional (*F–H*) and cross-sectional views (*I–K*) comparing DMSO-treated cysts (*F*, *I*, and *L*) with compound C1-treated (*G*, *J*, and *M*) and FSK-treated (*H*, *K*, and *N*) cysts. Compared with the DMSO control condition (*A*), compound C1 (*B*) and FSK (*C*) conditions exhibited a significant increase in the overall level of TFEB and nuclear enrichment within cysts. Cross-sectional views revealed a significant increase in the overall cytoplasmic and nuclear TFEB in treated conditions (*J* and *K*) compared with the distinct apically oriented perinuclear distribution of TFEB in the control condition (*I*). *L–N*: enlarged images of *insets* in which the outlines of the cystic epithelium are included for the purpose of signal context and clarity. Scale bars = 20 µm; scale bar in *insets* = 10 µm. Images are representative of 15 cysts acquired from each of 3 separate experiments. ****P < 0.0001.

To ascertain whether nuclear Tfeb translocation itself is sufficient to promote cystogenesis, MDCK cells were grown in a collagen matrix were used (21, 22). The adenylyl cyclase activator forskolin was used as a positive control to induce cyst growth (23, 24) and compared with compound C1, a curcumin derivative that has been shown to directly bind to Tfeb and induce its nuclear translocation independent of mTOR pathway modulation (25). MDCK cells were cultured for 5 days before incubation with 1 or 5 µM compound C1 or 10 µM forskolin, which were added daily before the acquisition of phase-contrast images on *day 9*. A qualitative increase in cyst diameter in both compound C1-treated (Fig. 9, *B* and *C*) and forskolin-treated (Fig. 9*D*) cyst cultures compared with those treated with DMSO was readily apparent (Fig. 9*A*). Direct measurement of cyst diameter confirmed a significant increase in cyst size following compound C1 exposure (Fig. 9*E*). Immunostaining of cyst cultures in three-dimensional and cross-sectional views revealed an increase in overall Tfeb staining as well as nuclear staining (Fig. 9, *F*–*N*) in both compound C1- and forskolin-treated cultures relative to DMSO-treated control cultures.

### TFEB Expression and TFEB Target Genes in Human ADPKD Tissue

**Figure 10.**
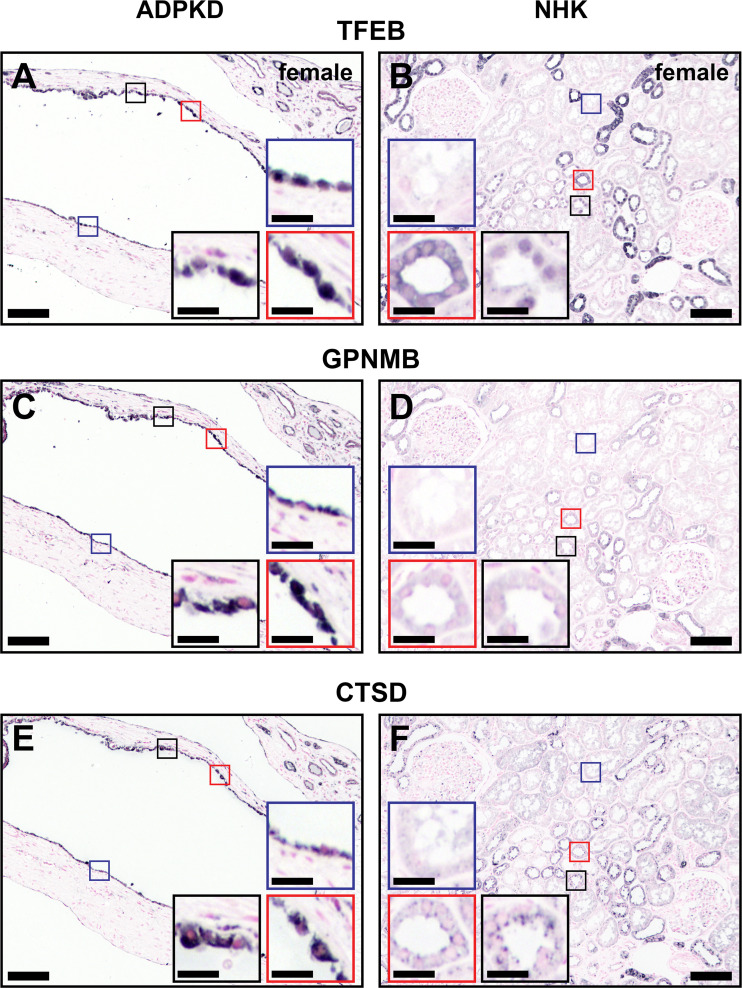
Transcription factor EB (TFEB) distribution in normal and autosomal dominant polycystic kidney disease (ADPKD) human renal tissues. Serial formalin-fixed paraffin-embedded sections derived from human ADPKD (*A*, *C*, and *E*) and normal human renal (*B*, *D*, and *F*) tissue were subjected to immunohistochemistry using antibodies raised against TFEB (*A* and *B*), glycoprotein nonmetastatic melanoma protein B (GPNMB; *C* and *D*), or cathepsin D (CTSD; *E* and *F*). Compared with normal renal samples (*B*), a significant increase in TFEB expression was observed in cyst-lining epithelial cells (*A*) and there was evidence of nuclear translocation. The TFEB downstream targets GPNMB and CTSD were significantly increased in human renal cysts (*C* and *E*) compared with normal renal samples (*D* and *F*). Scale bars = 100 µm; scale bars in *insets* = 20 µm. Images are representative of five ×10 fields within each genotype. NHK, normal human kidney.

To study the relevance of Tfeb in human ADPKD, the expression and subcellular localization of TFEB and two of its known targets, GPNMB and CTSD, were determined in serial FFPE sections derived from human patients with ADPKD and normal human kidney samples. Renal cysts exhibited strong staining of TFEB with evidence of nuclear translocation (Fig. 10*A* and Supplemental Fig. S7), which colocalized with the nuclear marker Hoechst 33342 (Supplemental Fig. S8, *A–C*). In normal renal tubules, TFEB expression was variable with some tubules exhibiting cytoplasmic staining, some nuclear staining, and most tubules with no apparent staining (Fig. 10*B* and Supplemental Fig. S8, *D–F*). GPNMB staining was significantly higher in renal cysts (Fig. 10*C*) that were TFEB positive (Supplemental Fig. S9) compared with normal human renal tubules (Fig. 10*D*). CTSD expression was also significantly upregulated in renal cysts (Fig. 10*E*) compared with normal human renal tubules (Fig. 10*F*) and correlated with renal cystic epithelial cell nuclear TFEB staining (Supplemental Fig. S10).

## DISCUSSION

This study reports that nuclear TFEB translocation characterizes cyst-lining epithelia in several models of polycystic disease, a pathway normally associated with nutrient deprivation and stress. Important findings include the following. First, nuclear TFEB localization was a binary response, mostly observed in cystic but not noncystic epithelia. mTORC1 activation, as assessed by phospho-S6 staining, was sporadically present in cystic and noncystic epithelia, suggesting that TFEB translocation did not directly parallel the activity of mTORC1 and suggesting that increased mTORC1 activity may not be a primary driving force in renal cyst development when contrasted with the observed ubiquitous nature of TFEB nuclear translocation. TFEB translocation was an early event, notably observed in the *Pkd1* KO mouse, where it preceded the appearance of tubular dilation. Nuclear TFEB translocation was also sustained in this model, present in *postnatal day 150* kidneys.

**Figure 11.**
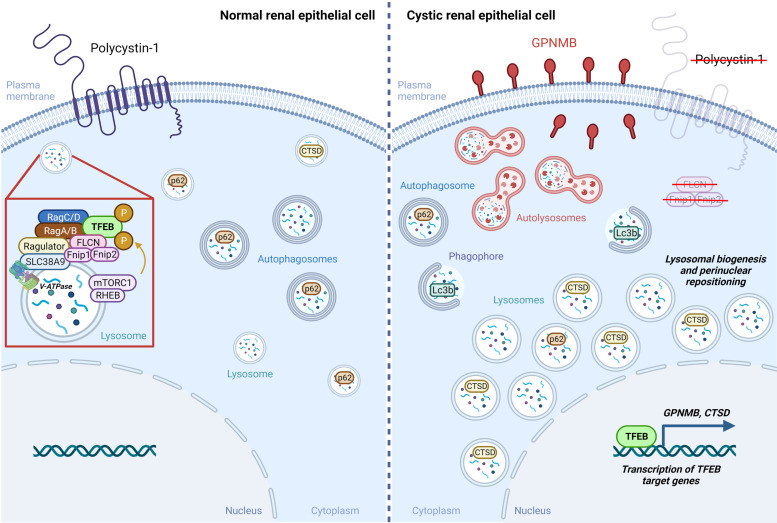
Summary of observed transcription factor EB (Tfeb)-related differences between normal and cystic renal epithelial cells. In normal renal epithelial cells (*left*), Tfeb is associated with the lysosomal compartment in the cytoplasm as a component of a large multiprotein complex (*left*, *inset*). In cystic epithelial cells (*right*) that develop due to the absence of polycystin-1, folliculin (Flcn), or the folliculin-interacting protein 1 and 2 (Fnip1/2) complex, there is a pronounced nuclear accumulation of Tfeb leading to the transcription of Tfeb target genes, such as glycoprotein nonmetastatic melanoma protein B (*GPNMB*) and cathepsin D (*CTSD*). In cells that lack polycystin-1 (*Pkd1*) expression, cytoplasmic light chain 3B (Lc3b) puncta increase in number, indicative of autophagy induction, and p62 puncta are decreased, indicative of increased degradation and supported by an observed increase in autophagic flux. Consistent with a role of the Tfeb pathway in lysosomal biogenesis and positioning, lysosomes increase in size and undergo perinuclear repositioning in the absence of *Pkd1*. In human ADPKD samples, upregulation and nuclear translocation of TFEB in renal cysts is associated with a concomitant increase in GPNMB and CTSD expression. This figure was created with BioRender.com.

Second, functional responses associated with nuclear TFEB translocation were observed in these models. These included the transcription and expression of Tfeb-associated gene products (Gpnmb and CTSB), lysosomal biogenesis, perinuclear lysosome distribution, and increased autophagic flux. Importantly, nuclear Tfeb was observed following the deletion of *Pkd1*, suggesting an undefined link between loss of polycystin-1 and TFEB translocation (Fig. 11).

Third, the TFEB agonist C1 caused enhanced cyst formation of MDCK cells in three-dimensional culture and was associated with nuclear Tfeb translocation. The magnitude of C1-induced cyst growth was comparable to that observed with forskolin. Forskolin-stimulated cyst growth was also marked by nuclear Tfeb translocation in the three-dimensional MDCK model consistent with a pathway linking adenylate cyclase to nuclear Tfeb translocation, albeit with a nuclear distribution that was distinct from compound C1.

Fourth, nuclear TFEB was observed in cyst-lining epithelia of human PKD tissue. Immunostaining of CTSD and GPNMB, two TFEB regulated gene products, was also increased. Collectively, these findings identify TFEB as a potentially important pathway for cystogenesis.

TFEB has been characterized as a master regulator of lysosomal biogenesis based on its central role in coordinating the expression of lysosomal hydrolases, membrane proteins, and autophagy-related genes. The subcellular localization of TFEB is regulated by phosphorylation at specific serine residues. In the present model, phosphorylation of TFEB by mTORC1 at Ser^211^ results in binding to 14-3-3 proteins and sequestration in the cytosol. The FLCN-FNIP1/2 complex plays a role in the recruitment of mTOR to the lysosome surface, where the complex functions as the GAP of RagC/D. Multiple other kinases have been reported to phosphorylate TFEB, including glycogen synthase kinase-3β, ERK, Akt, and protein kinase C (26). TFEB phosphorylation by these other kinases may account for the observed dissociation of nuclear TFEB distribution from mTORC1 activity in the PKD models studied.

Napolitano et al. (27) have identified an alternative mechanism that might explain the dissociation of mTORC1 phosphorylation S6 kinase from that of TFEB in the setting of hyperactivation of mTORC1 due to loss of folliculin activity. They observed that the constitutive activation of TFEB is a primary driver of the renal abnormalities in the folliculin KO mouse through increased expression of RagC and RagD GTPases. In this setting, mTORC1 promotes S6 kinase activity but not TFEB phosphorylation and cytosolic sequestration.

TFEB dephosphorylation precedes nuclear transport and requires calcineurin and lysosomal Ca^2+^ release through TRP channel mucolipin 1 (TRPML1). TFEB is marked by its translocation from the cytoplasm to the nucleus, where it regulates the transcription of genes associated with a palindromic sequence (5′- TCACGTGA-3′) near the transcriptional start site referred to as the CLEAR sequence (11). Several hundred genes have been identified as TFEB regulated.

The regulation of nuclear egress is also important in TFEB shuttling between the nucleus and cytosol but is less well understood. mTOR-dependent rephosphorylation is one mechanism for nuclear export (28). Phosphorylated TFEB is shuttled from the nucleus to the cytosol under the control of the exportin CRM1 (29). TFEB activity and cellular localization are also regulated by the E3 ubiquitin ligase STIP1 homology and U box-containing protein 1 (STUB1) (30) and by the histone acetyltransferase GCN5 (31).

The canonical pathway for TFEB activation is commonly associated with nutrient deprivation (32, 33). The aberrant regulation of renal metabolism in PKD as marked by aerobic glycolysis (Warburg effect) has been recognized as characteristic of renal cystic epithelia and recently has been the focus of strategies to inhibit cyst growth by targeting AMP kinase with agents such as metformin (4, 34). Aerobic glycolysis is distinct from the cellular response to nutrient deprivation. The latter is generally viewed as a means for insuring cell survival. A potential explanation linking aerobic glycolysis to TFEB activation is the recent observation that folliculin inhibits lactate dehydrogenase A. Predictably, loss of folliculin activity would promote a Warburg-type phenotype and result in inhibition of nuclear TFEB egress (35). Folliculin has also been localized to the cilia, where it controls mTORC1 signaling in response to flow stress (36).

A noncanonical role for TFEB has also been proposed as important in cancer with the identification of an association between TFEB and renal oncogenesis. Associations include BHD syndrome and gene fusions involving TFE3 and TFEB (37). However, loss of functional folliculin in these settings is not typically characterized by renal cystic disease but rather by tumors such as oncocytomas. It remains to be determined how increased nuclear TFEB promotes cystogenesis and whether the current canonical or noncanonical schemas will clarify the underlying mechanism.

This study raises several significant questions. First, does TFEB activity play a causal role in PKD? If nuclear sequestration of TFEB is either sufficient or necessary for cystogenesis, as has been demonstrated in a *Flcn* KO model (27), then would targeting TFEB cytosolic dephosphorylation or nuclear phosphorylation present reasonable therapeutic strategies for PKD? Alternatively, are specific TFEB functions observed in cystic epithelia required for cystogenesis? TFEB-dependent gene products, exemplified by CTSB and GPNMB, may play previously unrecognized roles in cystogenesis and growth. GPNMB, for example, is proproliferative and triggers the expression of matrix metalloproteinases (38).

Corollary questions include the following. Are the biogenesis and perinuclear redistribution of lysosomes necessary for cystogenesis? Would inhibition of either lysosomal biogenesis, fusion, or trafficking inhibit the growth of cysts? Is TFEB-mediated autophagy required for cyst growth? Recent work has highlighted a role for lysosomal movement and positioning in multiple cellular functions (39). The dysregulation of the temporal-spatial distribution of lysosomes observed with loss of *Pkd1* appears to be a novel finding.

Is the efficacy of therapies that have been or are currently under investigation for the treatment of PKD hindered or aided by their effects on TFEB activity? Rapamycin should promote TFEB translocation to the nucleus secondary to mTORC1 inhibition and thus stimulate cyst growth (40). However, rapamycin also directly and specifically binds to the lysosomal Ca^2+^ channel, TRPML1, and induces nuclear translocation of TFEB independent of its established role as an mTOR inhibitor (41). In the folliculin and folliculin-interacting protein KO models, mTORC1 activity did not directly correlate with nuclear TFEB distribution on an intercyst cell or intracyst basis despite the prior observation that phospho-S6 (Ser^235/236^) is increased in whole kidney lysates (10, 27). AMP-activated protein kinase has received attention as a potential therapeutic target for PKD (42, 43). However, metformin, an activator of AMP-activated protein kinase, has been recently reported to phosphorylate TFEB and increase its activity, a pharmacological response contrary to the intended strategy (44). In the *Flcn* KO model, it has been reported that AMP-activated protein kinase activity appears not to play a role in the renal cystic phenotype (27).

Finally, if nuclear TFEB characterizes cystic epithelia, then could specific TFEB-mediated gene products be used as biomarkers for cyst burden in patients with PKD? The identification of cyst epithelium-specific markers would potentially provide a valuable tool for monitoring disease progression and therapeutic interventions. GPNMB, CTSB, and CTSD, all known to be secreted proteins, have not been previously associated with PKD.

### Perspectives and Significance

Although PKD has long been recognized as a disorder of aberrant renal epithelial cell growth and differentiation, the role of nutrient-sensing signaling pathways, particularly those associated with lysosomal function, has not been well studied. The nuclear transcription factor TFEB links nutrient sensing at the lysosome to important functional responses at the cellular level. The recognition that TFEB-mediated responses characterize renal cystic epithelia provides new avenues for understanding cytogenesis, novel druggable targets, and biomarker discovery.

## DATA AVAILABILITY

Data will be made available upon reasonable request.

## SUPPLEMENTAL DATA

10.6084/m9.figshare.21843195.v2 Supplemental Fig. S1: https://doi.org/10.6084/m9.figshare.21843195.v2.

10.6084/m9.figshare.21843198.v2 Supplemental Fig. S2: https://doi.org/10.6084/m9.figshare.21843198.v2.

10.6084/m9.figshare.21843201.v2 Supplemental Fig. S3: https://doi.org/10.6084/m9.figshare.21843201.v2.

10.6084/m9.figshare.21843204.v1 Supplemental Fig. S4: https://doi.org/10.6084/m9.figshare.21843204.v1.

10.6084/m9.figshare.21843207.v2 Supplemental Fig. S5: https://doi.org/10.6084/m9.figshare.21843207.v2.

10.6084/m9.figshare.21843210.v2 Supplemental Fig. S6: https://doi.org/10.6084/m9.figshare.21843210.v2.

10.6084/m9.figshare.21843213.v2 Supplemental Fig. S7: https://doi.org/10.6084/m9.figshare.21843213.v2.

10.6084/m9.figshare.21843216.v2 Supplemental S8: https://doi.org/10.6084/m9.figshare.21843216.v2.

10.6084/m9.figshare.21843219.v2 Supplemental Fig. S9: https://doi.org/10.6084/m9.figshare.21843219.v2.

10.6084/m9.figshare.21843222.v2 Supplemental Fig. S10: https://doi.org/10.6084/m9.figshare.21843222.v2.

10.6084/m9.figshare.21843234.v2 Supplemental Table S1: https://doi.org/10.6084/m9.figshare.21843234.v2.

## GRANTS

Funding for this study was through a research endowment to the Shayman laboratory from the University of Michigan Medical School to support the study of lysosomal storage disorders. This work was supported by National Institutes of Health Grants DK106912 and P30CA168524. Research reported in this publication was supported by the National Cancer Institute under Award Number P30CA046592 using the following Rogel Cancer Center Shared Resource: Tissue and Molecular Pathology.

## DISCLOSURES

J.A.S. receives royalty income from patents licensed to Sanofi/Genzyme by the University of Michigan. Funding for this work was the result of an endowment for royalties received by the University of Michigan for the discovery and development of eliglustat tartrate by J.A.S. None of the other authors has any conflicts of interest, financial or otherwise, to disclose.

## AUTHOR CONTRIBUTIONS

J.A.S. conceived and designed research; J.M.S. performed experiments; J.M.S. and J.A.S. analyzed data; J.M.S. and J.A.S. interpreted results of experiments; J.M.S. prepared figures; J.M.S. and J.A.S. drafted manuscript; J.M.S. and J.A.S. edited and revised manuscript; J.M.S. and J.A.S. approved final version of manuscript.

## References

[1] Vasileva VY, Sultanova RF, Sudarikova AV, Ilatovskaya DV. Insights into the molecular mechanisms of polycystic kidney diseases. Front Physiol 12: 693130, 2021. doi:10.3389/fphys.2021.693130. 34566674 PMC8456103

[2] Bergmann C, Guay-Woodford LM, Harris PC, Horie S, Peters DJM, Torres VE. Polycystic kidney disease. Nat Rev Dis Primers 4: 50, 2018. doi:10.1038/s41572-018-0047-y. 30523303 PMC6592047

[3] Blair HA. Tolvaptan: a review in autosomal dominant polycystic kidney disease. Drugs 79: 303–313, 2019. doi:10.1007/s40265-019-1056-1. 30689194

[4] Rowe I, Chiaravalli M, Mannella V, Ulisse V, Quilici G, Pema M, Song XW, Xu H, Mari S, Qian F, Pei Y, Musco G, Boletta A. Defective glucose metabolism in polycystic kidney disease identifies a new therapeutic strategy. Nat Med 19: 488–493, 2013. doi:10.1038/nm.3092. 23524344 PMC4944011

[5] Napolitano G, Ballabio A. TFEB at a glance. J Cell Sci 129: 2475–2481, 2016. doi:10.1242/jcs.146365. 27252382 PMC4958300

[6] Calcagni A, Kors L, Verschuren E, De Cegli R, Zampelli N, Nusco E, Confalonieri S, Bertalot G, Pece S, Settembre C, Malouf GG, Leemans JC, de Heer E, Salvatore M, Peters DJ, Di Fiore PP, Ballabio A. Modelling TFE renal cell carcinoma in mice reveals a critical role of WNT signaling. eLife 5, 2016. doi:10.7554/eLife.17047. 27668431 PMC5036965

[7] Daccord C, Good JM, Morren MA, Bonny O, Hohl D, and Lazor R. Birt-Hogg-Dube syndrome. Eur Respir Rev 29: 200042, 2020. 32943413 10.1183/16000617.0042-2020 PMC9489184

[8] Baba M, Furihata M, Hong SB, Tessarollo L, Haines DC, Southon E, Patel V, Igarashi P, Alvord WG, Leighty R, Yao M, Bernardo M, Ileva L, Choyke P, Warren MB, Zbar B, Linehan WM, Schmidt LS. Kidney-targeted Birt-Hogg-Dube gene inactivation in a mouse model: Erk1/2 and Akt-mTOR activation, cell hyperproliferation, and polycystic kidneys. J Natl Cancer Inst 100: 140–154, 2008. doi:10.1093/jnci/djm288. 18182616 PMC2704336

[9] Chen J, Futami K, Petillo D, Peng J, Wang P, Knol J, Li Y, Khoo SK, Huang D, Qian CN, Zhao P, Dykema K, Zhang R, Cao B, Yang XJ, Furge K, Williams BO, and Teh BT. Deficiency of FLCN in mouse kidney led to development of polycystic kidneys and renal neoplasia. PLoS One 3: e3581, 2008. 18974783 10.1371/journal.pone.0003581 PMC2570491

[10] Hasumi H, Baba M, Hasumi Y, Lang M, Huang Y, Oh HF, Matsuo M, Merino MJ, Yao M, Ito Y, Furuya M, Iribe Y, Kodama T, Southon E, Tessarollo L, Nagashima K, Haines DC, Linehan WM, Schmidt LS. Folliculin-interacting proteins Fnip1 and Fnip2 play critical roles in kidney tumor suppression in cooperation with Flcn. Proc Natl Acad Sci USA 112: E1624–E1631, 2015. doi:10.1073/pnas.1419502112. 25775561 PMC4386336

[11] Sardiello M, Palmieri M, di Ronza A, Medina DL, Valenza M, Gennarino VA, Di Malta C, Donaudy F, Embrione V, Polishchuk RS, Banfi S, Parenti G, Cattaneo E, Ballabio A. A gene network regulating lysosomal biogenesis and function. Science 325: 473–477, 2009. doi:10.1126/science.1174447. 19556463

[12] van der Lienden MJC, Gasper P, Boot R, Aerts JMFG, van Eijk M. Glycoprotein non-metastatic protein B: an emerging biomarker for lysosomal dysfunction in macrophages. Int J Mol Sci 20: 66, 2018. doi:10.3390/ijms20010066. 30586924 PMC6337583

[13] Raben N, Puertollano R. TFEB and TFE3: linking lysosomes to cellular adaptation to stress. Annu Rev Cell Dev Biol 32: 255–278, 2016. doi:10.1146/annurev-cellbio-111315-125407. 27298091 PMC6490169

[14] von Manteuffel SR, Dennis PB, Pullen N, Gingras AC, Sonenberg N, Thomas G. The insulin-induced signalling pathway leading to S6 and initiation factor 4E binding protein 1 phosphorylation bifurcates at a rapamycin-sensitive point immediately upstream of p70s6k. Mol Cell Biol 17: 5426–5436, 1997. doi:10.1128/MCB.17.9.5426. 9271419 PMC232392

[15] Settembre C, Di Malta C, Polito VA, Garcia Arencibia M, Vetrini F, Erdin S, Erdin SU, Huynh T, Medina D, Colella P, Sardiello M, Rubinsztein DC, Ballabio A. TFEB links autophagy to lysosomal biogenesis. Science 332: 1429–1433, 2011. doi:10.1126/science.1204592. 21617040 PMC3638014

[16] Zhang W, Li X, Wang S, Chen Y, Liu H. Regulation of TFEB activity and its potential as a therapeutic target against kidney diseases. Cell Death Discov 6: 32, 2020. doi:10.1038/s41420-020-0265-4. 32377395 PMC7195473

[17] Tanida I, Ueno T, Kominami E. LC3 and autophagy. Methods Mol Biol 445: 77–88, 2008. doi:10.1007/978-1-59745-157-4_4. 18425443

[18] Yoshii SR, Mizushima N. Monitoring and Measuring Autophagy. Int J Mol Sci 18: 1865, 2017. doi:10.3390/ijms18091865. 28846632 PMC5618514

[19] Chauhan S, Ahmed Z, Bradfute SB, Arko-Mensah J, Mandell MA, Won Choi S, Kimura T, Blanchet F, Waller A, Mudd MH, Jiang S, Sklar L, Timmins GS, Maphis N, Bhaskar K, Piguet V, Deretic V. Pharmaceutical screen identifies novel target processes for activation of autophagy with a broad translational potential. Nat Commun 6: 8620, 2015. doi:10.1038/ncomms9620. 26503418 PMC4624223

[20] Pu J, Keren-Kaplan T, Bonifacino JS. A Ragulator-BORC interaction controls lysosome positioning in response to amino acid availability. J Cell Biol 216: 4183–4197, 2017. doi:10.1083/jcb.201703094. 28993468 PMC5716277

[21] Elia N, Lippincott-Schwartz J. Culturing MDCK cells in three dimensions for analyzing intracellular dynamics. Curr Protoc Cell Biol Chapter 4: 4.22.1–4.22.18, 2009. doi:10.1002/0471143030.cb0422s43. 19499508 PMC2786266

[22] Yuajit C, Homvisasevongsa S, Chatsudthipong L, Soodvilai S, Muanprasat C, Chatsudthipong V. Steviol reduces MDCK cyst formation and growth by inhibiting CFTR channel activity and promoting proteasome-mediated CFTR degradation. PLoS One 8: e58871, 2013. doi:10.1371/journal.pone.0058871. 23536832 PMC3594167

[23] Mangoo-Karim R, Uchic M, Lechene C, Grantham JJ. Renal epithelial cyst formation and enlargement in vitro: dependence on cAMP. Proc Natl Acad Sci USA 86: 6007–6011, 1989. doi:10.1073/pnas.86.15.6007. 2474825 PMC297761

[24] Taide M, Kanda S, Igawa T, Eguchi J, Kanetake H, Saito Y. Human simple renal cyst fluid contains a cyst formation-promoting activity for Madin-Darby canine kidney cells cultured in collagen gel. Eur J Clin Invest 26: 506–513, 1996. doi:10.1046/j.1365-2362.1996.177306.x. 8817166

[25] Song JX, Sun YR, Peluso I, Zeng Y, Yu X, Lu JH, Xu Z, Wang MZ, Liu LF, Huang YY, Chen LL, Durairajan SS, Zhang HJ, Zhou B, Zhang HQ, Lu A, Ballabio A, Medina DL, Guo Z, Li M. A novel curcumin analog binds to and activates TFEB in vitro and in vivo independent of MTOR inhibition. Autophagy 12: 1372–1389, 2016. doi:10.1080/15548627.2016.1179404. 27172265 PMC4968239

[26] Puertollano R, Ferguson SM, Brugarolas J, Ballabio A. The complex relationship between TFEB transcription factor phosphorylation and subcellular localization. EMBO J 37: e98804, 2018. doi:10.15252/embj.201798804. 29764979 PMC5983138

[27] Napolitano G, Di Malta C, Esposito A, de Araujo MEG, Pece S, Bertalot G, Matarese M, Benedetti V, Zampelli A, Stasyk T, Siciliano D, Venuta A, Cesana M, Vilardo C, Nusco E, Monfregola J, Calcagni A, Di Fiore PP, Huber LA, Ballabio A. A substrate-specific mTORC1 pathway underlies Birt-Hogg-Dube syndrome. Nature 585: 597–602, 2020. doi:10.1038/s41586-020-2444-0. 32612235 PMC7610377

[28] Napolitano G, Esposito A, Choi H, Matarese M, Benedetti V, Di Malta C, Monfregola J, Medina DL, Lippincott-Schwartz J, Ballabio A. mTOR-dependent phosphorylation controls TFEB nuclear export. Nat Commun 9: 3312, 2018. doi:10.1038/s41467-018-05862-6. 30120233 PMC6098152

[29] Li L, Friedrichsen HJ, Andrews S, Picaud S, Volpon L, Ngeow K, Berridge G, Fischer R, Borden KLB, Filippakopoulos P, Goding CR. A TFEB nuclear export signal integrates amino acid supply and glucose availability. Nat Commun 9: 2685, 2018. doi:10.1038/s41467-018-04849-7. 29992949 PMC6041281

[30] Sha Y, Rao L, Settembre C, Ballabio A, Eissa NT. STUB1 regulates TFEB-induced autophagy-lysosome pathway. EMBO J 36: 2544–2552, 2017. doi:10.15252/embj.201796699. 28754656 PMC5579343

[31] Wang Y, Huang Y, Liu J, Zhang J, Xu M, You Z, Peng C, Gong Z, Liu W. Acetyltransferase GCN5 regulates autophagy and lysosome biogenesis by targeting TFEB. EMBO Rep 21: e48335, 2020. doi:10.15252/embr.201948335. 31750630 PMC6945067

[32] Martina JA, Puertollano R. Rag GTPases mediate amino acid-dependent recruitment of TFEB and MITF to lysosomes. J Cell Biol 200: 475–491, 2013. doi:10.1083/jcb.201209135. 23401004 PMC3575543

[33] Martina JA, Diab HI, Brady OA, Puertollano R. TFEB and TFE3 are novel components of the integrated stress response. EMBO J 35: 479–495, 2016. doi:10.15252/embj.201593428. 26813791 PMC4772850

[34] Perrone RD, Abebe KZ, Watnick TJ, Althouse AD, Hallows KR, Lalama CM, Miskulin DC, Seliger SL, Tao C, Harris PC, Bae KT. Primary results of the randomized trial of metformin administration in polycystic kidney disease (TAME PKD). Kidney Int 100: 684–696, 2021. doi:10.1016/j.kint.2021.06.013. 34186056 PMC8801184

[35] Woodford MR, Baker-Williams AJ, Sager RA, Backe SJ, Blanden AR, Hashmi F, Kancherla P, Gori A, Loiselle DR, Castelli M, Serapian SA, Colombo G, Haystead TA, Jensen SM, Stetler-Stevenson WG, Loh SN, Schmidt LS, Linehan WM, Bah A, Bourboulia D, Bratslavsky G, Mollapour M. The tumor suppressor folliculin inhibits lactate dehydrogenase A and regulates the Warburg effect. Nat Struct Mol Biol 28: 662–670, 2021. doi:10.1038/s41594-021-00633-2. 34381247 PMC9278990

[36] Zhang Y, Liu Y, Dai Y, Ren Y, Bao G, Ai B, Jiang Y. Ciliary localization of folliculin mediated via a kinesin-2-binding motif is required for its functions in mTOR regulation and tumor suppression. FEBS Lett 595: 123–132, 2021. doi:10.1002/1873-3468.13959. 33064845 PMC7980781

[37] Kauffman EC, Ricketts CJ, Rais-Bahrami S, Yang Y, Merino MJ, Bottaro DP, Srinivasan R, Linehan WM. Molecular genetics and cellular features of TFE3 and TFEB fusion kidney cancers. Nat Rev Urol 11: 465–475, 2014. doi:10.1038/nrurol.2014.162. 25048860 PMC4551450

[38] Taya M, Hammes SR. Glycoprotein non-metastatic melanoma protein B (GPNMB) and cancer: a novel potential therapeutic target. Steroids 133: 102–107, 2018. doi:10.1016/j.steroids.2017.10.013. 29097143 PMC6166407

[39] Cabukusta B, Neefjes J. Mechanisms of lysosomal positioning and movement. Traffic 19: 761–769, 2018. doi:10.1111/tra.12587. 29900632 PMC6175085

[40] Saxton RA, Sabatini DM. mTOR signaling in growth, metabolism, and disease. Cell 169: 361–371, 2017. doi:10.1016/j.cell.2017.03.035. 28388417

[41] Zhang X, Chen W, Gao Q, Yang J, Yan X, Zhao H, Su L, Yang M, Gao C, Yao Y, Inoki K, Li D, Shao R, Wang S, Sahoo N, Kudo F, Eguchi T, Ruan B, Xu H. Rapamycin directly activates lysosomal mucolipin TRP channels independent of mTOR. PLoS Biol 17: e3000252, 2019. doi:10.1371/journal.pbio.3000252. 31112550 PMC6528971

[42] Song X, Tsakiridis E, Steinberg GR, Pei Y. Targeting AMP-activated protein kinase (AMPK) for treatment of autosomal dominant polycystic kidney disease. Cell Signal 73: 109704, 2020. doi:10.1016/j.cellsig.2020.109704. 32621956

[43] Caplan MJ. AMPK and polycystic kidney disease drug development: an interesting off-target target. Front Med (Lausanne) 9: 753418, 2022. doi:10.3389/fmed.2022.753418. 35174190 PMC8841847

[44] Paquette M, El-Houjeiri L, Zirden LC, Puustinen P, Blanchette P, Jeong H, Dejgaard K, Siegel PM, Pause A. AMPK-dependent phosphorylation is required for transcriptional activation of TFEB and TFE3. Autophagy 17: 3957–3975, 2021. doi:10.1080/15548627.2021.1898748. 33734022 PMC8726606

